# DeepWAS: Multivariate genotype-phenotype associations by directly integrating regulatory information using deep learning

**DOI:** 10.1371/journal.pcbi.1007616

**Published:** 2020-02-03

**Authors:** Janine Arloth, Gökcen Eraslan, Till F. M. Andlauer, Jade Martins, Stella Iurato, Brigitte Kühnel, Melanie Waldenberger, Josef Frank, Ralf Gold, Bernhard Hemmer, Felix Luessi, Sandra Nischwitz, Friedemann Paul, Heinz Wiendl, Christian Gieger, Stefanie Heilmann-Heimbach, Tim Kacprowski, Matthias Laudes, Thomas Meitinger, Annette Peters, Rajesh Rawal, Konstantin Strauch, Susanne Lucae, Bertram Müller-Myhsok, Marcella Rietschel, Fabian J. Theis, Elisabeth B. Binder, Nikola S. Mueller

**Affiliations:** 1 Department of Translational Research in Psychiatry, Max Planck Institute of Psychiatry, Munich, Germany; 2 Institute of Computational Biology, Helmholtz Zentrum München, Neuherberg, Germany; 3 Department of Neurology, Klinikum rechts der Isar, School of Medicine, Technical University of Munich, Munich, Germany; 4 German Competence Network Multiple Sclerosis (KKNMS), Klinikum rechts der Isar, Technical University of Munich, Munich, Germany; 5 Research Unit of Molecular Epidemiology and Institute of Epidemiology, Helmholtz Zentrum München, Neuherberg, Germany; 6 Institute of Epidemiology, Helmholtz Zentrum München, Neuherberg, Germany; 7 German Centre for Cardiovascular Research (DZHK), Berlin, Germany; 8 Munich Cluster for Systems Neurology (SyNergy), Munich, Germany; 9 Department of Neurology, St. Josef-Hospital, Ruhr-University Bochum, Bochum, Germany; 10 Munich Cluster for Systems Neurology (SyNergy), Munich, Germany; 11 Department of Neurology, University Medicine Mainz, Johannes Gutenberg University Mainz, Mainz, Germany; 12 NeuroCure Clinical Research Center, Department of Neurology, and Experimental and Clinical Research Center, Max Delbrück Center for Molecular Medicine, and Charitϩ –Universitätsmedizin Berlin, Berlin, Germany; 13 Department of Neurology with Institute of Translational Neurology, University of Münster, Münster, Germany; 14 Institute of Human Genetics, University Hospital Bonn and Division of Genomics, Life & Brain Research Centre, University of Bonn School of Medicine, Bonn, Germany; 15 Interfaculty Institute for Genetics and Functional Genomics, University Medicine and University of Greifswald, Greifswald, Germany; 16 Junior Research Group on Computational Systems Medicine, Chair of Experimental Bioinformatics, TUM School of Life Sciences Weihenstephan, Technical University of Munich, Freising-Weihenstephan, Germany; 17 Department I of Internal Medicine, Kiel University, Kiel, Germany; 18 Institute of Human Genetics, Helmholtz Zentrum München, Neuherberg, Germany and Institute of Human Genetics, Technical University of Munich, Munich, Germany; 19 Institute of Epidemiology and Social Medicine, University of Münster, Münster, Germany; 20 Institute of Genetic Epidemiology, Helmholtz Zentrum München, Neuherberg, Germany and Institute of Medical Informatics, Biometry, and Epidemiology, Chair of Genetic Epidemiology, Ludwig-Maximilians-Universität, Munich, Germany; 21 Institute of Translational Medicine, University of Liverpool, Liverpool, United Kingdom; 22 Department of Mathematics, Technical University of Munich, Garching, Germany; 23 Department of Psychiatry and Behavioral Sciences, Emory University School of Medicine, Atlanta GA, United States of America; National Center for Biotechnology Information (NCBI), UNITED STATES

## Abstract

Genome-wide association studies (GWAS) identify genetic variants associated with traits or diseases. GWAS never directly link variants to regulatory mechanisms. Instead, the functional annotation of variants is typically inferred by *post hoc* analyses. A specific class of deep learning-based methods allows for the prediction of regulatory effects per variant on several cell type-specific chromatin features. We here describe “DeepWAS”, a new approach that integrates these regulatory effect predictions of single variants into a multivariate GWAS setting. Thereby, single variants associated with a trait or disease are directly coupled to their impact on a chromatin feature in a cell type. Up to 61 regulatory SNPs, called dSNPs, were associated with multiple sclerosis (MS, 4,888 cases and 10,395 controls), major depressive disorder (MDD, 1,475 cases and 2,144 controls), and height (5,974 individuals). These variants were mainly non-coding and reached at least nominal significance in classical GWAS. The prediction accuracy was higher for DeepWAS than for classical GWAS models for 91% of the genome-wide significant, MS-specific dSNPs. DSNPs were enriched in public or cohort-matched expression and methylation quantitative trait loci and we demonstrated the potential of DeepWAS to generate testable functional hypotheses based on genotype data alone. DeepWAS is available at https://github.com/cellmapslab/DeepWAS.

## Author summary

In the era of steadily increasing amounts of available genetic data, we still lack novel and innovative ideas on how to improve fine-mapping of regulatory variants identified by genome-wide association studies (GWAS), especially in non-coding regions. Current approaches for the identification of functional variants conduct functional annotation after the GWAS analysis either using position-based overlaps of each variant with regulatory elements or deep-learning-based methods predicting regulatory effects per variant on cell-type-specific chromatin features. We here present DeepWAS, which integrates these regulatory effect predictions of single variants into a multivariate GWAS setting. Our results provide evidence that DeepWAS results directly identify disease/trait-associated SNPs with a common effect on a specific chromatin feature in a relevant tissue. We can show for multiple sclerosis, major depressive disorder, and body height, that the SNPs identified by DeepWAS are at least nominally significant in classical univariate GWAS analysis of the same cohorts or larger published GWAS. By integrating expression and methylation quantitative trait loci (eQTL and meQTL) information of multiple resources and tissues, we can show that DeepWAS identifies disease/trait-relevant transcriptionally active genomic loci. We demonstrate that DeepWAS identifies both known variants and highlights underlying molecular mechanisms.

## Introduction

Genome-wide association studies (GWAS) have been highly successful in identifying genetic variants associated with risk for common diseases and traits. However, going from pure association to mechanistic insight has been a much more challenging task. Identification of the true causal variants in the context of a disease or trait from within regions of associated variants is hampered by linkage disequilibrium (LD), as proximal variants are likely to co-segregate in a population. The functional variants can, in most cases, not be easily discerned within long stretches of such correlated DNA, which can span several genes and include hundreds of associated variants.

Additional post-processing approaches, so-called functional GWAS, have been introduced to provide missing functional annotation to classical GWAS [1]. The majority of published functional GWAS are based on the positional overlap of single-nucleotide polymorphisms (SNPs) with *cis*-regulatory elements such as promoters and enhancers (see Tak and Farnham [2] for a comprehensive review). These functional GWAS indicate that, for common diseases, the majority of associated SNPs reside in non-coding, regulatory regions [1]. One drawback of these methods is that the actual impact of each variant on regulatory elements is not assessed, as the annotation is based on positional overlap only. For example, two SNPs that localize to the same chromatin immunoprecipitation with a massively parallel sequencing (ChIP-seq) peak of a transcription factor (TF) might have the same, opposing, or no functional effects at all [3]. To resolve this shortcoming, *in silico* approaches predicting the degree of disruption of TF binding motifs have been used [4]. However, our understanding of the determinants of TF binding to known sequence motifs is still incomplete, limiting the success of such methods. Another post-processing approach to infer functional variants is the correlation of SNPs with gene expression or DNA methylation data in the form of expression and methylation quantitative trait locus (eQTL and meQTL) analyses [5]. Additional methods such as binding QTL (bQTL) studies for TF binding [6] or massively parallel reporter assays (MPRAs) now add experimental regulatory information on a single variant resolution [7], yet need to be performed in the cell type of interest. While these approaches can indicate regulatory effects of associated SNPs, they cannot identify single functional variants within an LD block.

Recent advances in systems genetics that harness the predictive power of deep learning might enhance the performance of functional SNP prioritization methods. The deep learning method “DeepSEA” uses only DNA sequence information to predict effects on regulatory chromatin features, such as histone marks, TF binding, or the presence of open chromatin [8]. For this annotation method, experimental, publicly available data from the ENCODE project [9] and the Roadmap Epigenomics Project [10] for cell type-specific TF binding, histone modifications, and chromatin states were used. This type of functional sequence annotation is superior to *post hoc*, position-based overlap methods, as it computes allele-specific differences regarding the effects of variants on regulatory elements and thus discerns SNPs with predicted functional impact in a given cell type from those just residing within an annotated element by chance. Importantly, the method allows for incorporating cell-type specific regulatory effects of variants at baseline as well as under experimentally challenged conditions, adding additional critical layers to understanding tissue- and context-specific disease mechanisms. This approach was further enhanced to predict tissue-specific gene expression levels and to prioritize putative causal variants associated with human traits and diseases [11]. Recently, deep learning was successfully applied to predict the impact of non-coding mutations in a family with autism spectrum disorder [12] and the clinical impact of single human mutations [13]. However, in all studies published to date, such deep learning-based annotations have only been applied *post hoc* to association results from classical GWAS.

We present a conceptually new approach fusing classical and functional GWAS. Instead of the classical approach analyzing single SNPs individually, before filtering them in a separate follow-up step for regulatory information, we here propose to first annotate SNPs for their regulatory potential, i.e., their affinity with a functional unit (FU) that is the combination of a specific chromatin feature, cell type, and treatment, before subjecting sets of SNPs with related functionality jointly to association tests by using regularized regression models. This approach is called multivariate FU-*W*ide *A*ssociation *S*tudy (DeepWAS). It reduces the multiple testing burden of classical GWAS and makes regulatory information on a single SNP level readily available without requiring a second analysis step. We made use of the most recent predictions for SNPs with regulatory potential, which will be constantly growing whenever new data becomes available in the future. For a proof of concept of DeepWAS, we used data from published GWAS of two common diseases, multiple sclerosis (MS) [14] and major depressive disorder (MDD) [15,16], as well as the quantitative trait of height [17], see Table 1. The heritability of MS and MDD was estimated to be 64% and 40%, respectively [18], comparable with other common diseases. The heritable nature of height is estimated to be between 70–90% [19]. GWAS meta-analyses have already identified 180 genetic loci for height [20], 200 for MS [21], and 102 independent loci for MDD [22]. We compared the results of DeepWAS in smaller samples to the results from GWAS meta-analyses of the same phenotypes. This allowed us to identify disease- or trait-associated FUs, generating novel supportive evidence for prior knowledge on pathophysiology. We also complemented DeepWAS results with QTL networks and generated novel testable hypotheses of disease mechanisms.

**Table 1:** Summary of sample sizes and genotype information for all cohorts.

						DeepWAS			
Phenotype	Data set	Cohort	Cases	Controls	Array	Total samples	Total analyzed SNPs	FU	dSNPs
MS	KKNMS	DE1	3,934	8,455	OMEX	15,283	7,968,337	637	53
		DE2	954	1,940	I660				
MDD	MDDC	recMDD	879	746	I550	3,627	8,768,555	237	61
		BoMa	597	1,292	I550				
height	KORA	S3	3,094	3,094	AFFY	5,866	8,460,286	381	43
		S4	2,772	2,772	AFFY				

MS = Multiple sclerosis, MDD = Major depressive disorder, OMEX = Illumina OmniExpress BeadChips, I660 = Illumina Human660W-Quad BeadChips, I550 = Illumina HumanHap550-Quad BeadChips and AFFY = Affymetrix Human SNP Arrays.

## Results

### Directly integrating regulatory information into genotype-phenotype associations: The DeepWAS approach

To enable the integration of regulatory information into the classical GWAS setting, regulatory information on a single SNP level is required. To retrieve a set of regulatory SNPs with effects on cell type-specific chromatin features, we employed DeepSEA SNP effect predictions [8]. DeepSEA is a deep learning-based method where the effect of specific chromatin feature (*e*.*g*., transcription factor (TF) binding) on a 1,000 bp sequence is predicted in the context of a cell type (*e*.*g*., GM10847), and a treatment (*e*.*g*., TNFα). We generated sequences of 1,000 bp regions centered around each GWAS SNP position (eight million SNPs) for the two different SNP alleles and used the pre-trained DeepSEA network to obtain allele-specific probabilities for each FU (*e*.*g*., the FU “GM10847:NFκB-TNFα” was informed by all ChIP-seq peaks of the TF NFκB treated with TNFα in the GM10847 cell line). For the present study, we used 919 pre-defined FUs (see S1 Table). We then employed the DeepSEA e-value metric that estimates the impact of a SNP on the functional readout by comparing the allele-specific probabilities per SNP to one million random SNPs from the 1,000 genomes project [23]. Only SNPs with significant e-values (e-value<5×10^−5^) were thus selected as likely to be regulatory. This filtering identified around 40,000 predicted regulatory SNPs, *i*.*e*., SNPs with effects in one FU. We next grouped SNPs that are predicted to moderate the same FU, resulting in 919 SNP sets. The rationale of this grouping on the level of FUs stems from the idea that the majority of available chromatin features were TFs (85%, see S1 Table) and that one TF influences a specific cellular function through accumulation of its downstream effects via multiple SNPs on a series of loci. This optimized SNP selection improves the power to identify sets of functional SNPs that may play a role in the etiology of the disease and maps them directly to a specific context–*i*.*e*., a cell type, transcription factor, or stimulation condition.

**Fig 1:**
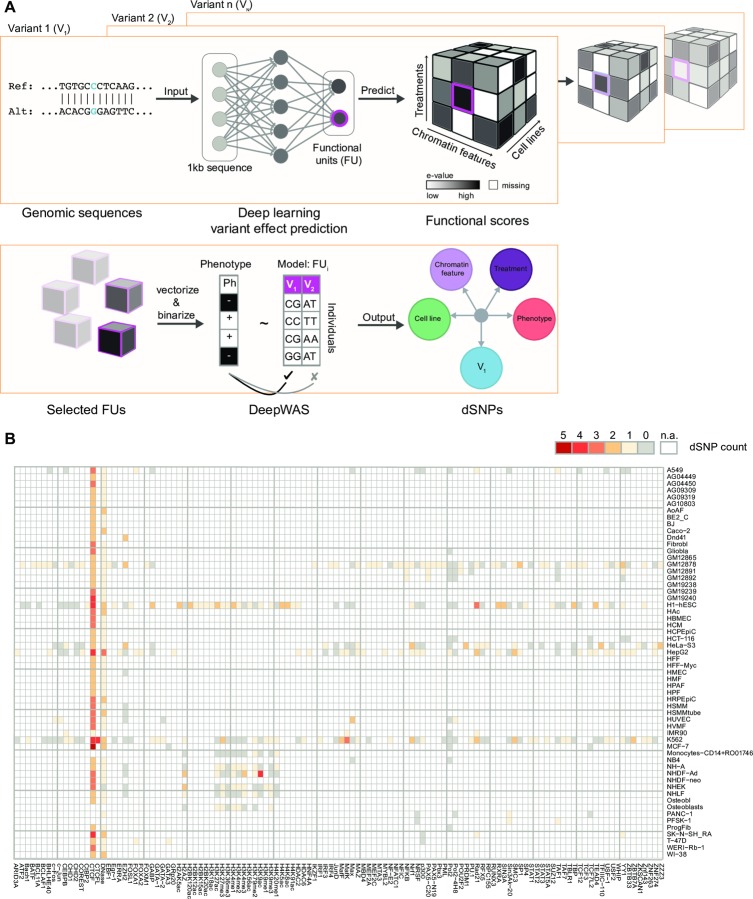
Workflow of DeepWAS. (**A**): A deep-learning based framework predicts combined binding probabilities for chromatin features, cell lines, and treatments, called functional units (FU) for 1,000 bp centered around a SNP. FUs are selected for a potential functional role of a variant using a cutoff for functional scores. This process is repeated for all genotyped variants. The genotype-phenotype association is analyzed for each FU using LASSO regression with stability selection. Unlike GWAS, DeepWAS implicates a regulatory mechanism underlying the phenotype of interest with information on relevant cell lines and TFs. (**B**): DeepWAS was applied to 36,409 regulatory SNPs that were retained after filtering for allele-specific effects in any given FU. These SNPs were tested for an association with multiple sclerosis (MS). The heatmap shows the number of selected chromatin features vs. cell lines. Chromatin features are limited to be present in at least two distinct cell lines. Missing values, represented in white, show FUs for which no data were available.

To finally associate these regulatory SNPs with a disease or trait in a multivariate manner, we employed L1-regularized (LASSO) linear regression per FU with its set of SNPs as predictors. By using LASSO, we were able to jointly analyze SNPs and consider the association of each SNP with the phenotype conditioned on all other SNPs. Especially with small cohort sizes, LASSO might lead to different results across different runs. To enhance robustness of the association results, we use the stability selection approach, that uses resampling to assess the stability of selected SNPs. We further refer to the approach of multivariate FU-*W*ide *A*ssociation *S*tudy as DeepWAS. From all 919 FU models used in this proof of concept study, we extracted the regulatory SNPs that showed a significant association and defined them as dSNPs (see Materials and Methods and Fig 1). The DeepWAS approach identified SNP-phenotype associations directly in a cell type-specific regulatory context.

### Application of DeepWAS

We conducted three genetic analyses on two case/control datasets and one population-based cohort using the DeepWAS approach: we compared individuals suffering from either MS or MDD to healthy controls in the case/control cohorts and analyzed the body height in the third sample (Table 1), using the covariates sex, age, cohort membership, and ancestry components to control for any remaining population substructure. First, we applied DeepWAS to an adequately-powered GWAS dataset for MS (KKNMS GWAS, 15,283 individuals from two independent MS case-control cohorts [14]) and found that 637 out of 919 FU models were associated with at least one variant (see Fig 1B). Most models identified one dSNP per FU, while 147 models identified two, three, or four dSNPs to jointly moderate a FU. In total, 53 unique MS-specific dSNPs, outside the major histocompatibility complex (MHC) region on chromosome 6, were identified by DeepWAS. These dSNPs moderate 120 chromatin features in 133 cell lines (see S2 Table). Note that 15 of the 53 dSNPs were in pairwise LD with each other (*r*^*2*^≥0.5), indicating that we identified 38 independent loci. 111 MS-specific dSNPs mapped to the MHC region. In addition to MS, we analyzed underpowered GWAS data sets for MDD (3,627 individuals recruited for recurrent MDD [15,16]) and height (5,866 individuals of the population-based KORA cohort [17]). Sixty-one MDD-specific dSNPs in 237 FUs (S1A Fig and S3 Table) and 43 height-specific dSNPs in 381 FUs (S1B Fig and S4 Table) were identified in these DeepWAS.

### Loci identified by DeepWAS are concordant with loci found in cohort-matched GWAS

**Fig 2:**
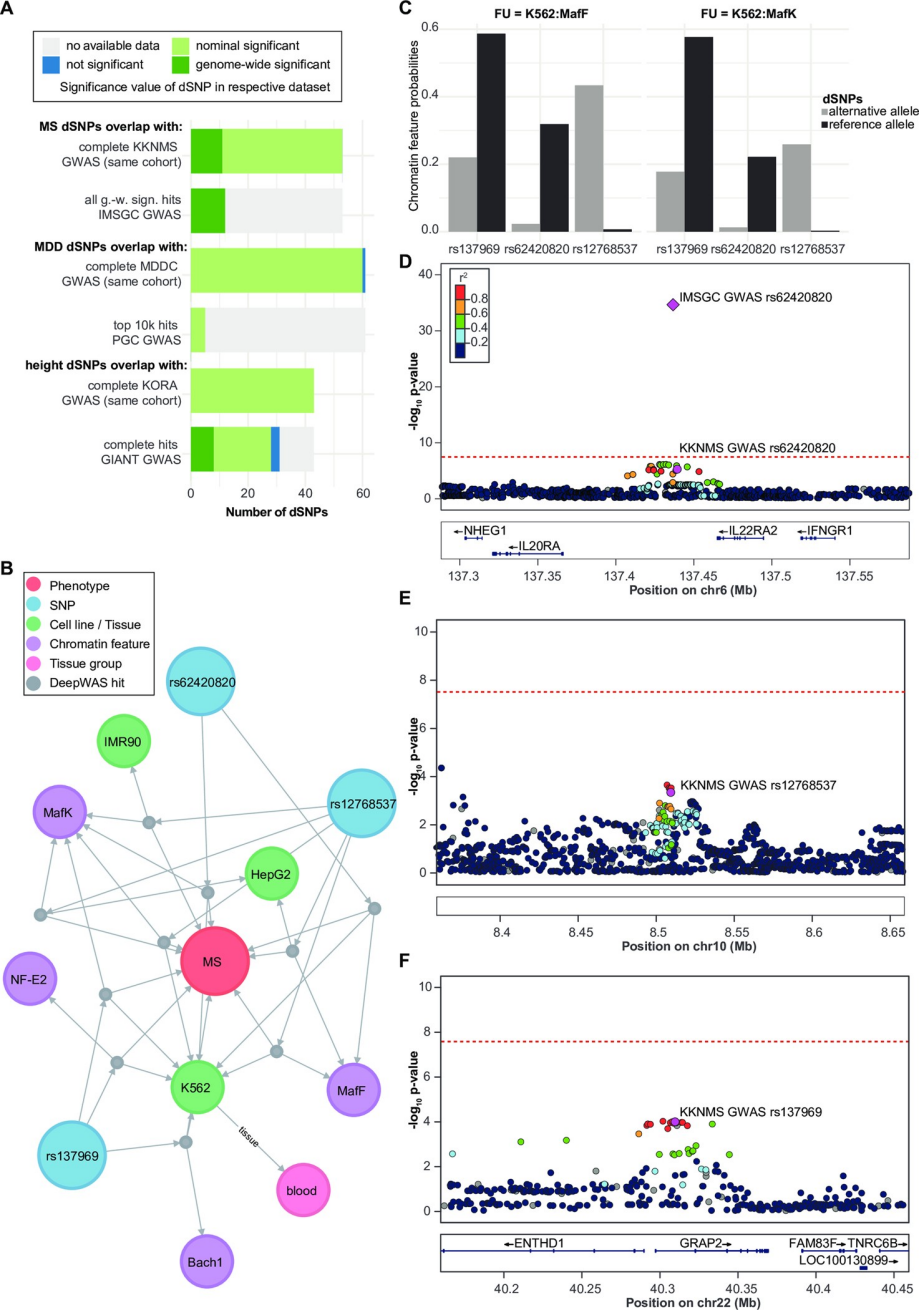
Comparison of DeepWAS vs. GWAS results. (**A**): Bar plot of the overlap of cohort-matched GWAS and consortia GWAS SNPs with dSNPs. g.-w. s = genome-wide significant. (**B**): Network of MS-specific dSNPs generated by using a graph database and showing the dSNP rs62420820 in the K562 cell line, a genome-wide significant signal in the IMSGC MS GWAS, but sub-threshold in the cohort-specific KKNMS GWAS. Edges represent the association relation of dSNPs, chromatin features with or without treatment, cell lines, and top-level tissue group. (**C**): Bar plots showing the predicted DeepSEA probabilities for dSNP sequences carrying the *alternative* and reference allele group by their FU. (**D-F**): Locus-specific Manhattan plots of the MS-specific dSNPs rs62420820, rs12768537, and rs137969, based on classical GWAS. Plots were produced using LocusZoom (https://github.com/statgen/locuszoom) with EUR samples of the 1,000 genomes November 2014 reference panel on the hg19 build. Dots represent KKNMS GWAS *p*-values and the diamond shows the IMSGC GWAS signal *p*-value. Color of the dots indicates LD with the lead variant = dSNP (magenta), grey dots have LD *r*^*2*^ missing.

To evaluate the convergence of DeepWAS and GWAS, (multi-SNP *vs*. single-SNP approaches), we evaluated how many dSNPs mapped to cohort-specific results from classical GWAS and to results of larger published meta-analysis of GWAS (Fig 2A). In the published GWAS of the KKNMS dataset used in the present study, variants in the MHC region, as well as variants at 15 loci outside of this region, were significantly associated with MS on a genome-wide level [14]. Among these 15 loci 7 have at least one regulatory SNP. When comparing KKNMS GWAS and DeepWAS results on a single SNP level, eleven of the 53 MS-specific dSNPs or their LD proxies (*r*^*2*^≥0.5) mapped to six independent loci (*CLEC16A*, *MAZ*, *EVI5*, *CD58*, *SHMT1*, and an intergenic region on chromosome 10). The remaining dSNPs (*n* = 42) showed an association strength with at least nominal significance in the original GWAS with *p*-values ≤5.13×10^−4^, but did not reach genome-wide significance. To estimate the predictive power of DeepWAS *vs*. GWAS, we first partitioned the KKNMS data into a training set (80%) and a test set (20%). Next, DeepWAS models were fitted without stability selection on all putative regulatory SNPs and GWAS with logistic regression models for each genome-wide significant KKNMS GWAS hit. We then calculated the area under the curve (AUC) of the regression models for each SNP. The DeepWAS predictors had AUCs ranging from 0.59–0.66 (mean = 0.608 ± 0.009) in the test set, with the best predictor involving two variants. GWAS AUCs ranged between 0.59–0.62 (mean = 0.601 ± 0.007) in the test set. For the SNPs that were dSNPs and reached genome-wide significance in the KKNMS GWAS (n = 11), we directly compared the AUC values. Only one SNP had a lower maximal AUC in our DeepWAS approach (multi-SNP model) as compared to GWAS (single SNPs), indicating that, for 91% of dSNPs in common with GWAS hits, prediction accuracy was higher using DeepWAS.

For MDD, 60 out of the 61 MDD-specific dSNPs or their proxies (*r*^*2*^≥0.5) reached nominal significance in a univariate GWAS from the same cohort (MDDC *n* = 3,627 individuals, no genome-wide significant association).

All of the 43 height-specific dSNPs reached nominal significance in the classical GWAS in the same cohort (KORA cohort *n* = 5,866 individuals, one genome-wide significant association) with max. *p*-values ≤7.7×10^−3^.

The DeepWAS approach was, therefore, able to identify and validate disease relevant loci. It is possible that these loci were too weak to be detected by cohort-matched GWAS. The observation that these loci nevertheless showed strong signals in DeepWAS, may indicate that DeepWAS takes advantage of the fact that conditional correlations of relevant SNPs with the phenotype are often more significant than marginal correlations. However, future, larger case/control cohorts need to be analyzed to fully assess this possibility.

### DeepWAS validates loci identified by other GWAS

To further validate our results, we compared loci identified by DeepWAS with GWAS loci identified by larger studies. The aim was to validate dSNPs in a separate GWAS. We used the GWAS dataset of the International Multiple Sclerosis Genetics Consortium (IMSGC [24]) that included over 47,000 MS cases (~10 times more cases than the KKNMS dataset) and 68,000 controls, and which identified 200 non-MHC genome-wide risk loci. We found that 129 of these variants showed at least nominal significance in the cohort-matched GWAS and that 39 of the loci contained at least one regulatory SNP. Here, a total of 15 dSNPs mapped to ten independent genome-wide significant GWAS loci: *CLEC16A*, *EPS15L1*, *EVI5*, *CD58*, *LINC00271* as well as intergenic regions on chromosome 5 (nearby genes: *LOC100505625* and *PAPD7)*, chromosome 10 (nearby genes: *ZNF438* and *ZEB1-AS1*), chromosome 22 (nearby genes: *ENTHD1* and *GRAP2)*, chromosome 11 (nearby genes: *DRAP1* and *TSGA10IP*), and chromosome 6 (nearby genes: *IL20RA* and IL22RA2). Eight of these 15 dSNPs, corresponding to 4 out of 10 loci, were also genome-wide significant in the KKNMS GWAS (*CD58*, *EVI5*, *CLEC16A* and an intergenic region on chromosome 10). The top 10,000 IMSGC discovery stage GWAS SNPs contained 23 dSNPs, which represents a 144-fold enrichment (permutation *p*<0.001) over randomly sampled regulatory SNP sets (*n* = 36,409 regulatory SNPs).

No MDD-specific dSNPs overlapped with genome-wide significant MDD loci, but four dSNPs were among the top 10,000 SNPs of the currently largest GWAS for MDD by the PGC and UK BIOBANK [22] (which included 246,363 MDD cases and 561,190 controls and identified 102 genome-wide significant loci with eight loci showing nominal significance in the cohort-matched GWAS; PGC GWAS dSNP max. *p*-value ≤2.8×10^−4^), which represents a 2.3-fold enrichment (permutation *p*-value = 0.094) over randomly sampled regulatory SNP sets (*n* = 31,929 regulatory SNPs) and their LD proxies. These four dSNPs mapped to four independent loci: *LARP6-LRRC49*, two intergenic regions on chromosome 7 (nearby genes: *WNT2* and *ASZ1*, *and ATG9B* and *ABCB8*) and a locus on chromosome 1 (nearby genes: *C1orf220* and *MIR4424*).

Eight of the 43 height-specific dSNPs mapped to seven independent genome-wide significant loci (*DIS3L2*, *ZBTB38*, *LCORL*, *PDLIM4*, *ZNF311*, *HABP4*, and *PXMP4*) of the latest GWAS from the GIANT Consortium, which included over 183,727 individuals and identified 180 genome-wide significant loci [20], with 40 loci nominally significant in the KORA data. Eight dSNPs were among the top 10,000 GIANT GWAS SNPs, which represents a 32-fold enrichment (permutation *p*<0.001) over randomly sampled regulatory SNP sets (*n* = 34,661) regulatory SNPs.

In all the three DeepWAS, this approach identified regulatory SNPs that overlapped not only with SNPs associated with the same traits in the cohort-matched GWAS, but also with genome-wide significant associations from the larger consortia GWAS for these traits. We thus demonstrate that DeepWAS is powerful for detecting associations that did not reach genome-wide significance in the smaller cohort-matched GWAS but could be validated in larger studies.

### Deriving hypotheses on disease-associated mechanisms in MS from DeepWAS results

We next wanted to illustrate how DeepWAS can accelerate the discovery of disease mechanisms. Within the DeepWAS results for MS, we identified, for example, the intergenic region *IL20RA-IL22RA2* on chromosome 6 that includes dSNP rs62420820 (Fig 2B), which was genome-wide significant in the IMSGC GWAS (*p*-value = 9.26×10^−36^, Fig 2D) and nominally significant in the KKNMS GWAS conducted on the MS cohort used for the DeepWAS analysis (*p*-value = 1.23×10^−5^, Fig 2D). In comparison to the published GWAS-based results, DeepWAS adds the novel and testable hypothesis that the TFs MafF and MafK contribute to MS susceptibility. The IMSGC GWAS and dSNP rs62420820 shows allele-specific TF binding differences for MafF and MafK in the leukemia cell line K562 (Fig 2C). Of note, additional dSNPs were identified within the FUs MafK:K562 (Fig 2B–2E: dSNP rs12768537 on chromosome 10 and Fig 2B–2F: dSNP rs137969 on chromosome 22) and MafF:K562 (dSNP rs12768537), supporting a role of these TFs in the etiology of MS.

We also identified dSNPs that were detected at a genome-wide significance level by both the cohort-matched KKNMS and the IMSGC GWAS. This included dSNP rs1985372 on chromosome 16, located in the *CLEC16A* locus, previously suggested as a candidate gene for MS (the dSNP rs1985372 was significant in the KKNMS GWAS on the same cohort [14] and is in complete LD with the SNP rs2286974 (*r*^*2*^ = 0.99), which was reported in the IMSGC GWAS[24]). DeepWAS now adds the regulatory information that these SNPs alter TF binding of GABP, GATA-1, GATA-2, p300, STAT1, STAT2, STAT5A, and TBLR1, all expressed in K562 cells, and that these TFs play a MS-specific role in the regulation of *CLEC16A*.

### Characterization of dSNPs

**Fig 3:**
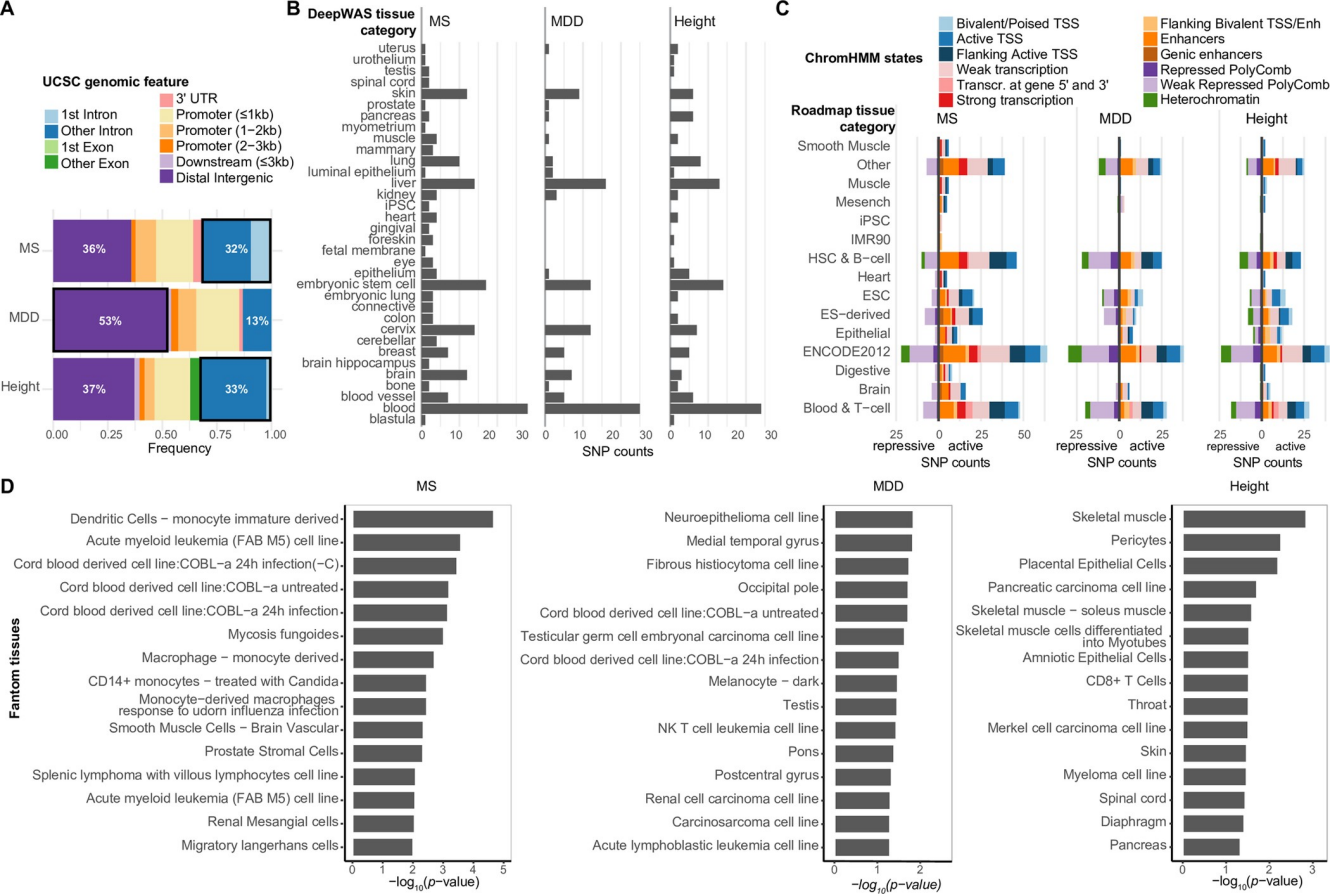
Functional characterization of DeepWAS hits. (**A**): Annotation of the genomic regions in which dSNPs are located: 63–87% of the genomic positions of dSNPs overlapped with non-coding DNA elements. Seventeen of 53 MS-specific (32%), 14 of 43 height-specific (33%) and 8 of 61 MDD-specific (13%) dSNPs mapped to introns (first and other introns). Over a half of the MDD-specific dSNPs (53%) resided in distal intergenic regions (>3 kb). None of the MS- and MDD- specific dSNPs were located in exons. (**B**): Bar plots for each phenotype showing the number of unique dSNPs annotated to a top-level tissue category (ENCODE). (**C**): Overlap of MS-, MDD-, and height-specific dSNPs with ChromHMM states from Roadmap epigenomes based on top-level tissue group matching. Most of our MS- and height-specific dSNPs mapped to predicted active chromatin states (82–86%), whereas nearly half of MDD-specific dSNPs mapped to inactive chromatin states (43%). (**D**) Tissue enrichment with FANTOM gene expression data. The top 15 significantly enriched tissues are shown (all *p*-values≤0.05).

We followed these results up by further characterizing the identified dSNPs from the three independent analyses. DSNPs for MS and height were more likely to be located in intronic regions (32–33% dSNPs in first or other introns) while dSNPs for MDD were more likely to cluster in distal intergenic regions (>3 kb = 53% vs. >3 kb = 36–37% for MS and height, respectively). MS- and MDD-specific dSNPs were never found within coding regions (Fig 3A).

DSNPs were always identified in a cell-type specific manner. We thus interrogated the cell type specificity on the level of tissue category, in order to reduce complexity (Fig 3B). Note that tissue categories were influenced by the richness of investigated cell types per category. For example, the tissue group blood encompassed 79 cell types, while brain contained only 14 tissues. Overall, more tissue groups were identified when the number of identified dSNPs increased. Interestingly, compared to MS and MDD, a lower number of identified dSNPs for height were relevant in brain tissues. At the same time, a larger number of height dSNPs were active in pancreatic tissue. Notably, an association of height with pancreatic cancer has previously been shown [25].

As DeepWAS includes only a limited number of histone marks, we next overlapped dSNPs with predicted chromatin states from the 15-state ChromHMM model [26] (Fig 3C). For both DeepWAS cell types (ENCODE) and 111 epigenomes, we used top-level tissue categories to overlap dSNPs with chromatin state predictions in the respective matched tissues. We observed tissue- and context- (disease or trait) specific roles of chromatin states. Most of the MS-specific dSNPs mapped to active chromatin states (82%, see Fig 3C), suggesting that they play a role in regulating transcriptional activities and active processing of RNA and DNA. For MDD-specific dSNPs, a larger fraction overlapped with repressive marks (43%, see Fig 3C), indicating an important role for silencing gene functions.

To investigate the tissue specificity of the genomic loci around dSNPs and to extend the number of disease-relevant tissues, we further tested if our dSNPs and their proxies (*r*^*2*^≥0.5) were enriched in the loci of genes expressed in a tissue-specific manner by leveraging Fantom CAGE data [27]. MS-specific dSNPs were significantly enriched in the regions active in different immune cell types, height-specific dSNPs in skeletal muscle cells, pericytes, and also a pancreatic carcinoma cell line, and MDD-specific dSNPs in neurocytoma as well as in different brain regions and immune cells (*p*-value ≤0.05, see Fig 3D).

### Regulatory effect of MS-specific dSNP loci

**Fig 4:**
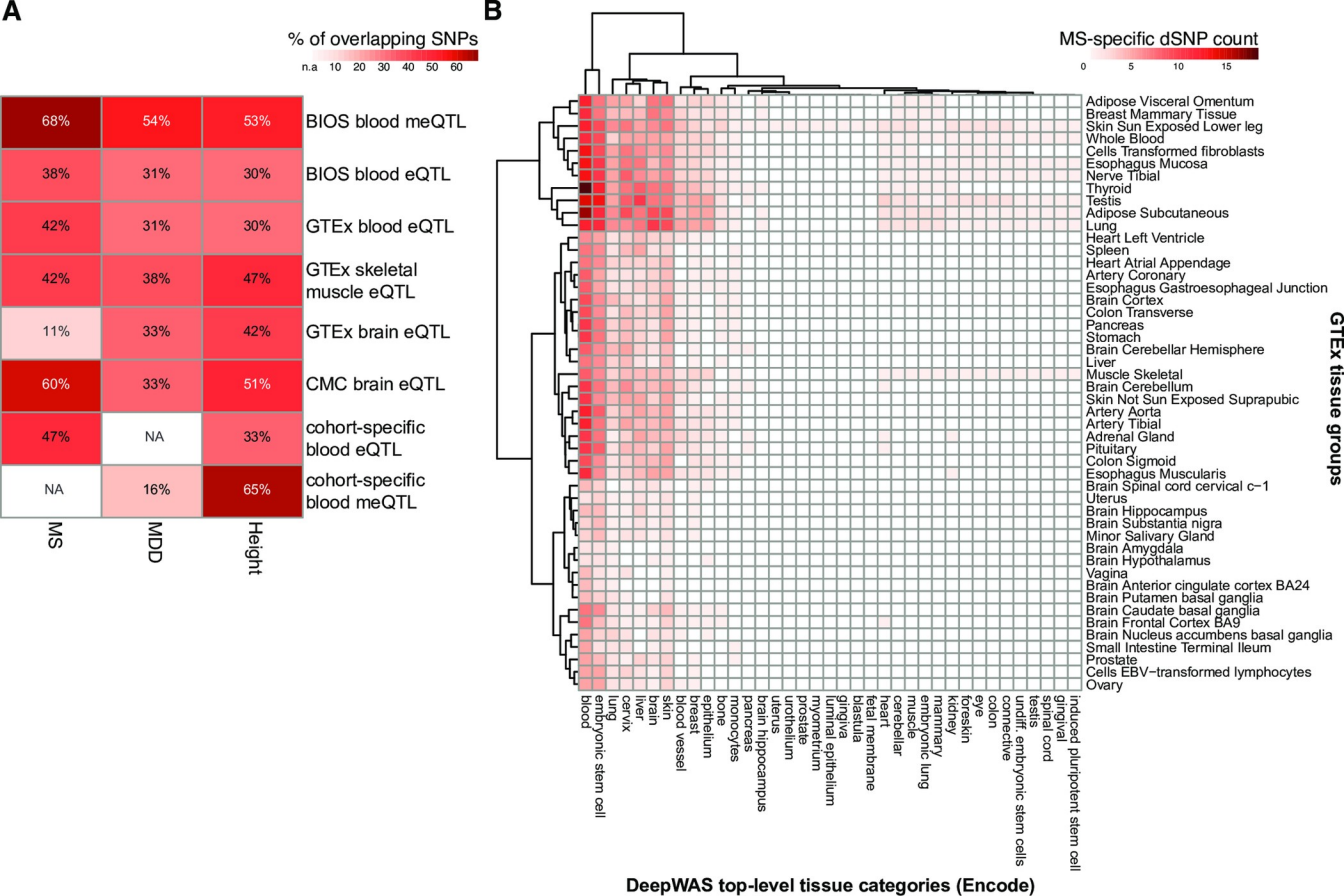
Context-related regulatory capacity of dSNPs. (**A**): Heatmap showing the percentage of overlap of MS-, MDD-, and height-specific dSNPs or their proxies (*r*^*2*^≥0.5) with *cis*-meQTL and *cis*-eQTL data from multiple resources, see also S2–S4 Tables. (**B**): Heatmap depicting GTEx tissue groups and DeepWAS top-level tissue category overlap among the MS-specific dSNP FUs.

Allele-specific effects on chromatin features and TF binding are likely to be reflected by changes in DNA methylation and gene expression. To test whether the MS-specific dSNPs or their proxies (*r*^*2*^≥0.5) were associated with differences in gene expression and DNA methylation, we used publicly available *cis*-meQTL, *cis*-eQTL, and *cis-*eQTM data from multiple resources: 1) The Biobank-Based Integrative Omics Study (BIOS [28]) analyzing whole blood (>2000 samples), the CommonMind Consortium (CMC [29]) dorsolateral prefrontal cortex data (DLPFC) (n = 603 samples), and GTEx data [30]. In the largest resource, BIOS, we observed that 36 of the 53 non-MHC, MS-specific dSNPs were significant meQTLs (68%) and 20 significant eQTLs (38%), which represents a 1.7- and 1.9-fold enrichment over randomly sampled regulatory SNPs and their assigned LD proxies, respectively (permutation *p*-values≤0.011, see Fig 4A and S2 Table). We next restricted DeepWAS hits to dSNPs moderating immune cell lines (n = 13 dSNPs, 46 chromatin features, 4 cell lines and 50 FUs, S2 Table), given the relevance of this tissue in MS [21]. Of this subset of 13 immune MS-specific dSNPs, 62% (n = 8 dSNPs) overlapped with meQTL variants (meSNP) and 38% (n = 5 dSNPs) with eQTL variants (eSNP) in BIOS. In their recent study, the IMSGC identified significant eQTL effects in naive CD4+ T cells and monocytes for only 18% of their significant GWAS loci [21]. When overlapping our immune MS-specific dSNPs with the same QTL data sets, we found 43% (n = 23 SNPs) of these dSNPs to be part of eQTLs in CD4+ T cells and 38% (n = 20 dSNPs) in monocytes. In addition to publicly available data, we also conducted an eQTL analysis using blood gene expression levels from a subset of 319 MS patients of the MS cohort. In total, 47% (n = 25 dSNPs) of the MS-specific dSNPs showed a significant eQTL effect in this data (Fig 4A and S2 Table), of which 14 dSNPs had previously been identified as part of eQTLs in blood. In the GTEx database, we found 42% of these dSNPs to have a significant effect on blood eQTLs (S2 Table).

Taken together, QTL data from various resources support that the DeepWAS of MS-identified SNPs reside in transcriptionally relevant regions, emphasizing their putative regulatory role in immune function. Moreover, we applied Combined Annotation Dependent Depletion (CADD) [31] to identify pathogenic variants. Seven MS-specific dSNPs showed PHRED scores >10, indicating seven pathogenic variants (13%). For all regulatory MS SNPs (n = 36,298), we found 4,898 pathogenic SNPs (13%).

### Regulatory effects of MDD and height dSNP loci

The 61 MDD- and 43 height-specific dSNPs were also transcriptionally active in the respective relevant tissues (see Fig 4A and S3 and S4 Tables) and tagged more BIOS eQTLs and meQTLs than expected by randomly sampling of regulatory SNPs and assigning their LD proxies (MDD: fold enrichment for eQTLs = 1.7 and meQTLs = 1.5, permutation *p*-value = 0.009 and 0.015, respectively; height: fold enrichment for eQTL = 1.5 and meQTLs = 1.5, permutation *p*-value = 0.068 and 0.013, respectively, see Materials and Methods). For a subset of both the MDDC and the KORA cohort, methylation levels and/or expression levels were measured. We, therefore, calculated meQTL and eQTL effects and found 16% of MDD-specific dSNPs with significant meQTL effects, 65% of the height-specific dSNPs with meQTL and 33% with eQTL effects (see Fig 4A and S3 and S4 Tables).

### Disease mechanisms on the level of functional units

While DeepWAS can be used to predict the phenotype from the genotype, it is also interesting to annotate the relationship of FUs to a disease or trait. For example, MS is an immune-mediated disorder affecting the central nervous system (CNS). Naturally, the CNS is difficult to directly examine as a study tissue. DeepWAS might here be used to identify a good proxy study tissue.

**Fig 5:**
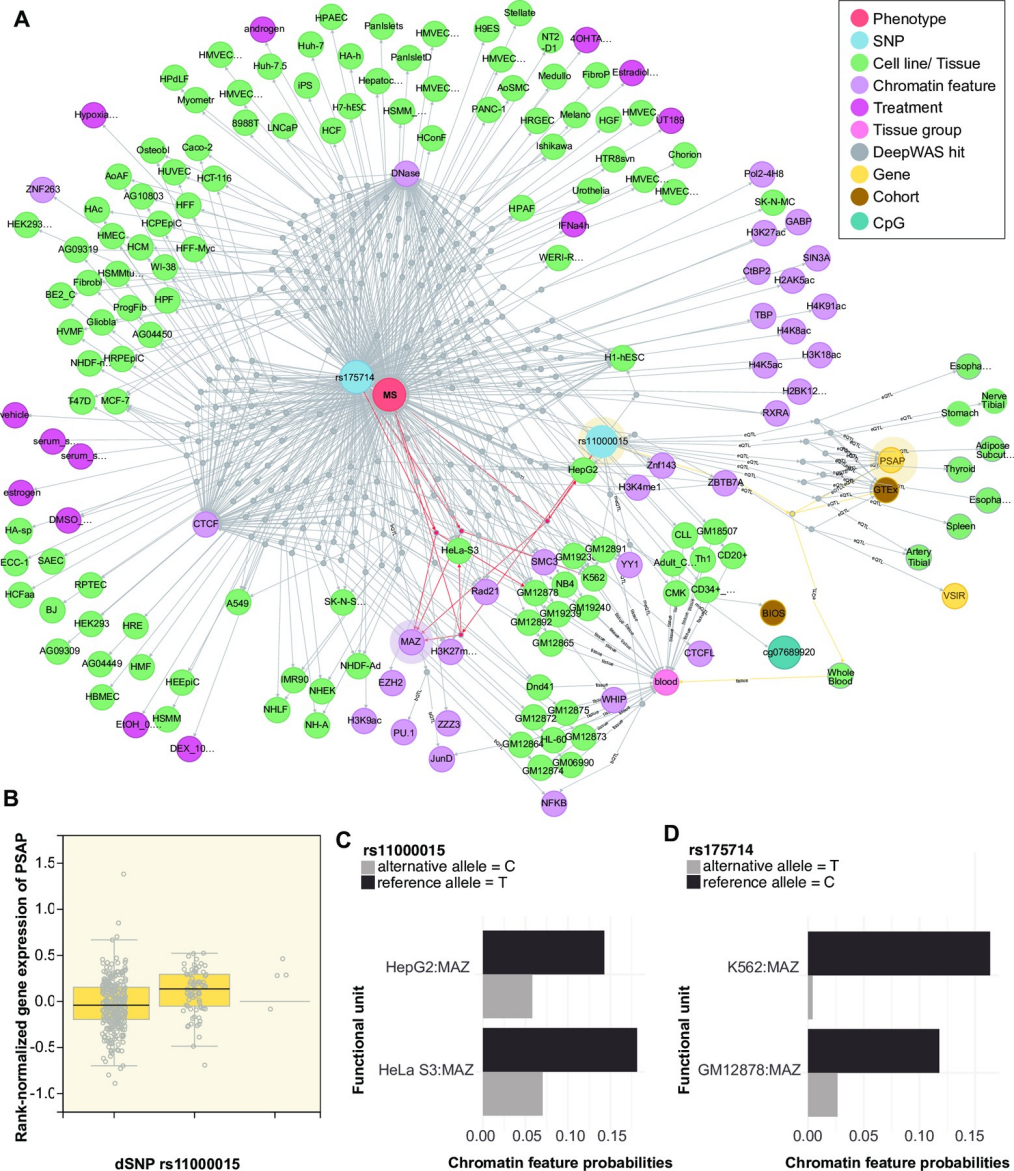
QTL network. (**A**) Network showing one of the putative key regulators for MS, dSNP rs175714 on chromosome 14. DSNP rs175714 is associated with differential TF binding of the TF MAZ, one of the top-associated loci in the KKNMS GWAS, where no significant transcriptional effect could be identified in the *post hoc* analysis. Edges represent the associations between dSNPs and chromatin features with or without treatment, cell lines, top-level tissue group, CpGs, and genes through dummy nodes identified either using DeepWAS or QTLs. Dummy nodes are used for preserving all entities of dSNP and QTL associations. Edges highlighted in red show the DeepWAS results for MAZ, in yellow show the eQTL connections illustrated in B, and shades refer to downstream QTL results shown in B. (**B**) Box plot of GTEx whole blood eQTL data showing the relationship between *PSAP* gene expression and dSNP rs11000015 genotype. (**C-D**) Chromatin feature probabilities for the significant FU of the dSNP sequences carrying the reference (black) and alternative (gray) allele.

Moreover, DeepWAS results can be used to identify single SNPs as key regulators, *i*.*e*., SNPs with effects on multiple chromatin features. DeepWAS identified, for example, the intergenic dSNP rs175714 on chromosome 14 as a key regulator for MS (Fig 5A and 5B). It affects the binding of multiple chromatin features at the same time (n = 29) in 116 cell types (Fig 5A and 5B). One of these chromatin features is the TF MAZ. MAZ itself is one of the top-associated loci of the KKNMS GWAS (tag SNP rs34286592 on chromosome 16, *p*-value = 4.58×10^−10^), but no significant transcriptional effect was previously identified in a *post hoc* analysis of the GWAS [14]. Interestingly, the MS-specific dSNP rs175714, together with the MS-specific dSNP rs11000015 on chromosome 10, had a significant effect on the binding of MAZ and they were jointly associated with MS disease status. The dSNP rs11000015 was correlated with expression levels of the Prosaposin (*PSAP*) gene in multiple tissues, whole blood gene expression levels of PSAP are shown in Fig 5C. *PSAP* codes for Prosaposin, a precursor of several small nonenzymatic glycoproteins termed sphingolipid activator proteins that assist in the lysosomal hydrolysis of sphingolipids [32]. Sphingolipids are the main components of nervous tissue and have been previously linked to MS [33]. Moreover, *PSAP* has been shown to be differentially expressed in the whole blood of MS patients compared to controls [34].

Another example is the TF MEF2C in the analysis of MDD, where DeepWAS identified the intergenic SNP rs7839671 on chromosome 8 as one of the key regulators for MDD (see S2 Fig). *MEF2C* is an important risk gene for MDD and the *MEF2C* gene itself is one of the top-associated loci of the PGC GWAS for MDD [22]. SNP rs7839671 and its proxies were associated with differences in mRNA expression of *SPIDR* and *MCM4* and were part of meQTLs with an intergenic region (for more details, see S2 Fig). The MEF2 TF family has been reported to play a major role in synaptic plasticity, which is thought to be disturbed in MDD, especially in the context of stress. Chen and colleagues [35] identified the TF MEF2 as a master regulator of developmental metaplasticity, which is important for guiding developmental structural and functional neuronal plasticity. Additional evidence was found by Barbosa *et al*.[36], relating MEF2 to activity-dependent dendritic spine growth and suggesting that this TF may suppress memory formation.

### QTL network analyses

In-depth investigation of the wealth of additional regulatory capacities of dSNPs were carried out by generating QTL networks that combine all pairwise links of meQTL (SNP-CpG), eQTL (SNP-gene), eQTM (CpG-gene), and dSNP-FU information. QTL network analyses helped us to identify the SNPs that showed joint effects on epigenetic and transcriptomic levels, *i*.*e*., dSNP = eSNP = meSNP where the dSNP harbors an eQTM. The resulting networks are called three-way QTL interaction networks. Three MS-specific dSNPs on chromosome 17, rs2273030, rs4925172 (both in complete LD with each other *r*^*2*^ = 1), and rs7207666 (*r*^*2*^ = 0.7) are located in the *SHMT1* locus, a candidate gene for MS [14,21] (S3A Fig). The genetic variants and *SHMT1* are connected via eQTL, meQTL, and eQTM relations (S3B Fig). In comparison to the previously published findings on the locus, DeepWAS informs that the TF Yin Yang 1 (YY1), expressed in multiple cell lines, may lead to a downregulation of *SHMT1* gene expression and hypermethylation of cg25492364 and cg26763362, thus connecting the disease-associated SNPs with specific regulatory functions.

Moreover, a three-way QTL interaction network analysis identified the height-specific dSNP rs7146599 on chromosome 14 to affect a cascade of eight chromatin features in eleven cell lines (see S4A Fig). The network also included rs2871960 on chromosome 3, linked to the *ZBTB38* locus and correlated with multiple CpG sites. *ZBTB38* has been previously shown to play an important regulatory role in height [37].

Thee-way QTL interaction network analysis identified the MDD-specific dSNP rs163105 on chromosome 5 to alter the expression of *SKIV2L2* (also known as *MTR4*). This gene has already been shown to be differentially expressed between depressed women and controls [38] (see S4B Fig). Furthermore, *SKIV2L2* has been implicated in the stress response and neurodegeneration through the nuclear exosome-targeting (NEXT) complex [39].

In summary, DeepWAS allows for the direct identification of putative master regulators, TFs and chromatin features, for subsequent in-depth analysis of genetic association signals.

## Discussion

In classical GWAS, the association of all SNPs is tested independently of each other on a genome-wide scale, thereby implicitly assuming that any SNP could affect the function of any cell state at any time. It is now clear that disease associations, especially for common disorders, are driven by SNPs that influence the function of regulatory elements. Hence, it is likely not necessary to test all SNPs in GWAS, instead, functional annotation could be used to prioritize putative risk variants. So far, several *post hoc* functional annotations of GWAS results have been reported [40]. Here, we present DeepWAS, a novel analysis tool for genetic associations that fuses classical GWAS and functional annotation into one single step (Fig 1). We employed the powerful deep learning-based method DeepSEA to predict regulatory effects of chromatin features in various cell types on a single SNP level. In addition, we implemented multi-SNP regression models with L1 regularization to identify so-called dSNPs. The SNPs of one FU were only selected when jointly associated with the disease or trait. To the best of our knowledge, this study is the first to combine deep learning-based predictors with multivariate models. By applying DeepWAS to three data sets, we showed that this method allows direct fine-mapping of GWAS associations at a single-base resolution and for direct functional annotation of the association signals to both chromatin features and cell types, generating novel mechanistic hypotheses. We also demonstrated that DeepWAS might increase the power to detect true positive signals, by pre-selecting functionally relevant SNPs and integrating multivariate statistics.

We applied DeepWAS to a well-powered GWAS dataset for MS (*n* = 15,283 individuals) and to an underpowered GWAS data for MDD (*n* = 3,627 individuals) and height (*n* = 5,866 individuals). In all three phenotypes, many SNPs in many genes contribute to genetic variation in the population and the effect size of each SNP outside the MHC region is small [41]. Therefore, large sample sizes are needed to discover additional risk variants. We identified 35 putative new candidate MS risk SNPs outside of the MHC region that did not reach genome-wide significance in GWAS (in total: 53 non-MHC, MS-specific dSNPs). For MDD, DeepWAS prioritized 57 new putative risk variants (in total: 61 MDD-specific dSNPs) and, for height, 35 new risk variants (in total: 43 height-specific dSNPs), even though the classical GWAS approach for MDD and height did not yield genome-wide significant results. Importantly, when comparing the dSNPs identified in these smaller cohort-matched GWAS to large consortia GWAS of the same phenotype, DeepWAS detected SNPs that reached genome-wide significance in these large consortia GWAS but not in smaller GWAS of the samples used for DeepWAS (Fig 2). Importantly, all dSNPs were associated at least with nominal significance in cohort-matched GWAS, with a subset of dSNPs reaching genome-wide significance. Interestingly, when comparing dSNPs to the larger consortia GWAS, DeepWAS detected SNPs that reached genome-wide significance in these GWAS but not in the smaller-scale GWAS on the individual cohorts (Fig 2). For example, 23% of the 53 MS-specific dSNPs were previously identified in the ISMGC MS GWAS including more than 135,000 individuals (*n*>47,000 MS cases). One-third of these dSNPs were not detected in the univariate KKNMS GWAS using the same cohorts as in DeepWAS (*n*>15,000 individuals).

By jointly analyzing SNPs, DeepWAS considers the correlation of each SNP with the phenotype, conditional on all other relevant SNP within an FU. This may increase the power to detect weak associations compared to GWAS (single SNP approach) because the conditional correlation of a SNP with the phenotype can often be substantially higher than the marginal correlation. DeepWAS, therefore, models the underlying biology more accurately, by explicitly modeling the polygenic architecture of complex phenotypes to account for the effects of multiple susceptibility loci. The increased power of DeepWAS is underlined by the observation that when, for example, all regulatory MS SNPs (*n* = 36,409 SNPs), without grouping to FUs, were used as input to a single LASSO regression model with stability selection, only 19 SNPs showed a significant association with MS (S5 Fig), of which four mapped outside the MHC region. This is in contrast to the 164 MS-specific dSNPs identified using DeepWAS, of which 53 were outside of the MHC region. Notably, these 36,409 SNPs resided in only 25,000 independent loci, which is also reflected by the fact that 16 out of 53 MS dSNPs were not independent of each other. The regulatory genotypes have, thus, little correlation, and can be subjected to L1 penalization. Additional penalization by the meta-parameter L2 (alpha) did not significantly improve the DeepWAS approach (S5 Fig) and is more difficult and time-consuming. In summary, DeepWAS can increase the power to detect associations of a phenotype with regulatory variants. Genome-wide significant consortia GWAS variants were detected using DeepWAS in smaller samples, where the variants only showed sub-threshold signals using classical GWAS. As outlined below, functional analyses of identified dSNPs suggest that the additional signals discovered using DeepWAS but not using classical GWAS reside within known, disease-relevant functional pathways and thus likely constitute true-positive associations.

Particularly for non-coding regulatory SNPs, DeepWAS has an advantage over GWAS followed by *post hoc* annotation and allows identifying transcriptionally relevant regions in the disease context (Figs 2–5). In fact, in all three DeepWAS, dSNPs were identified in cell types and enhancers previously shown to be relevant for the tested phenotype. For example, 47% of the MS-specific dSNPs (*n* = 35) affected the binding of chromatin features in hematopoetic tissue, and another 30% affected chromatin features in brain tissue or spinal cord (*n* = 16; Fig 3B). These findings highlighted that genetic disease risk is driven by the altered binding of chromatin features mainly in these two tissues.

DeepWAS results also pointed towards convergent regulatory mechanisms of specific TFs in both MS and MDD. For both disorders, DeepWAS identified a set of SNPs modulating binding of a TF that was found in GWAS to be associated with the disorders. This result suggests that SNPs associated with the gene encoding the TF and SNPs altering its binding to target transcripts jointly affect the phenotype. The gene *MAZ* on chromosome 16, for example, has been previously identified as a genome-wide significant GWAS locus for MS [14]. DeepWAS identified several dSNPs that include the TF MAZ in a FU (Fig 5). As a second example, we identified MDD-specific dSNPs altering the binding of TF MEF2C (S2 Fig). SNPs in the locus encoding MEF2C are the top signal in meta-analyses for major depression [22].

To support the validity of the predicted regulatory effects of our DeepWAS associations, we provided multiple lines of evidence that dSNPs and their surrounding loci are indeed functionally active in their respective tissue. We chose to prioritize DeepWAS results based on the additional regulatory impact of DNA methylation and gene expression, as shown by their meQTL and eQTL effects. DeepSEA, together with eQTL data, has been previously applied to prioritize disease-associated variants [11]. We observed that 68% of MS-specific dSNPs were meQTLs and 38% eQTLs in the largest QTL resource, BIOS. When using only random sets of regulatory SNPs with no disease association, no such overlap was found. For all investigated phenotypes, we observed a significant overlap between dSNPs and meQTL SNPs (Fig 4, fold enrichment ≥1.5 and permutation *p*-value over 1,000 random sets ≤ 0.015. For MDD- and MS-specific dSNPs, we found a significant overlap with eQTL SNPs (Fig 4, fold enrichment ≥1.5 and permutation *p*-value over 1,000 random sets ≤ 0.011). Moreover, as MS is a disorder developing in the peripheral immune system, we investigated if our dSNPs alter the expression of CD4+ T cells or influence the expression in monocytes, and found twice as many eQTL effects for dSNPs as described in the published IMSGC GWAS for MS (43% in CD4+ T cells and 38% in monocytes found using DeepWAS *vs*. 18% in both cell types in the IMSGC GWAS). Using expression profiles from GTEx, we found more MS-specific dSNPs for blood eQTL SNPs than in brain-related eQTL SNPs (42% *vs*.11%). This supports the theory that MS is most likely initially triggered by perturbation of immune responses, but that also the functional responses of brain cells are altered and may have a role in targeting an autoimmune process to the CNS.

Finally, we explored whether generating QTL interaction networks of our dSNPs and extracting the SNPs with an impact on methylation and expression, which also coincidently an eQTM, can help identify likely functional risk mechanisms. We identified dSNPs in the *SHMT1* gene, a published MS GWAS locus [14], where DeepWAS QTL network analysis pinpoints the TF Ying Yang 1 (YY1), expressed in multiple cell lines including immune related cells, as a potential novel risk factor (S3 Fig). YY1 is a ubiquitously expressed TF shown to be essential for B cell development [42] and serves as master regulator of T cell exhaustion [43]. Such dysregulation of immune related cells has been shown to promote MS progression [44]. Additional SNP-protein association studies (pQTLs) [45] that showed how the dSNP rs2273030 alters YY1 protein abundances could be extended to develop clinical applications in the context of MS.

DeepWAS is mostly limited by the comprehensiveness of regulatory element catalogues like ENCODE and Roadmap. ENCODE lacks, for example, information for a number of relevant disease-specific stimulation conditions as well as disease-related tissues. We have previously reported on the importance of testing SNPs in stimulated conditions, and shown that glucocorticoid response-moderating SNPs only become apparent in the stimulation condition and are not overlapping with baseline eQTLs [46]. Of note, the glucocorticoid receptor itself is central for the stress response and has been previously implicated in the pathogenesis of MDD [46]. In addition, data from cell lines or bulk tissues will miss variants with effects only on specific cell types, as well as cell type-specific effects dependent on the systemic, developmental, and/or tissue context. It is, therefore, important to be able to retrain the DeepSEA neural network with additional publicly available chromatin features and with newly generated experimental data. This will be possible using the DeepWAS pipeline publicly available at https://github.com/cellmapslab/DeepWAS.

In summary, our results indicate that DeepWAS, a method combining deep learning-based functional SNP annotation and considering a possible multivariate effect of SNPs to moderate a trait or disease, is a powerful tool to uncover disease mechanisms for common disorders and traits. It also allows direct identification of regulatory SNPs by having a single base resolution and not being limited by the LD structure of the locus, since the regulatory SNPs are mostly independent and because regulatory SNPs are analyzed jointly only if they are predicted to modulate the same FU. With ever-increasing amounts of available functional data, the DeepWAS approach will become even more valuable in the figure and will allow integration of both publicly as well as unpublished data generated by individual labs. DeepWAS is a versatile, publicly available tool that can be applied to any GWAS dataset in conjunction with the code available for DeepSEA. While we tested DeepWAS in small and medium-size samples and observed a potential increase in power in detecting phenotype-relevant functional SNPs, applying this method to very large data sets will be even more informative.

## Materials and methods

### Ethics statement

All responsible ethics committees have provided positive votes for the individual studies. All study participants gave written informed consent.

### Clinical samples

#### Major depressive disorder cohorts (MDDC)

Two MDD cohorts, recMDD and BoMa, were analyzed. The recMDD cohort consisted of 1,774 Caucasian individuals recruited at the Max-Planck Institute of Psychiatry (MPIP) in Munich, Germany and two satellite hospitals in the Munich metropolitan area (BKH Augsburg and Klinikum Ingolstadt): 756 controls (581 women, 275 men) and 879 cases diagnosed with recurrent major depression (585 women, 294 men). Please see Muglia *et al*. [15] for more details on sample recruitment and characterization. The BoMa cohort consisted of 1,889 Caucasian individuals: 1,292 controls (656 men, 636 women), 597 (212 men, 385 women) of which had a depressive disorder. Recruitment strategies and further characterization have been described previously [16].

#### Multiple sclerosis cohorts (KKNMS)

MS cohorts, referred to as DE1 and DE2, were analyzed. Both data sets included patients diagnosed with either MS or the prodromal clinically isolated syndrome. DE1 consists of 3,934 cases and DE2 consists of 954 cases; for more details see Andlauer *et al*. [14]. Controls for these cohorts were obtained from several cohorts across Germany, for more details see Andlauer *et al*. [14].

#### Population-based cohort (KORA)

The study population consisted of participants from the KORA (Kooperative Gesundheitsforschung in der Region Augsburg) study [17], which has been collecting clinical and genetic data from the general population in the region of Augsburg, Germany for more than 20 years. Here, the independent cohorts S3 (3,094 individuals) and S4 (2,772 individuals), including their follow-ups (F3 and F4), were analyzed.

### Genotype data

Genotype data was generated for each cohort individually, see Table 1. Details on the methods used can be found in the individual papers (recMDD [15], BoMa [16], KKNMS [14], and KORA [17]). Quality control (QC) of KKNMS genotype data and imputation have been previously described [14] and the same pipeline was applied for KORA and recMDD genotype data. The QC was conducted in PLINK 1.90b3s or higher (https://www.cog-genomics.org/plink2) for each cohort separately. QC steps on samples for KKNMS, KORA, and recMDD included removal of individuals with a missing rate >2%, cryptic relatives (*PI-HAT* >0.0125), an autosomal heterozygosity deviation (|*F*_*he*t_| >4 SD), and genetic outliers (distance in the ancestry components from the mean >4 SD). QC steps on variants included removal of variants with a call rate <98%, a MAF <1%, and HWE test *p*-values ≤10^−6^. Furthermore, variants on non-autosomal chromosomes were excluded. QC steps for BoMa included removal of variants with a call rate <95%, individuals with a missing rate >2%, an |*F*_*he*t_| <0.2, a difference in variant missingness between cases and controls <2%, variants with a MAF<1%, and variants with HWE test *p*-values ≤10^−6^ in controls or *p*-values ≤10^−10^ in cases. All non-autosomal chromosomes were excluded. Imputation was performed separately for each cohort with IMPUTE2 (https://mathgen.stats.ox.ac.uk/impute/impute_v2.html), following phasing in SHAPEIT (https://mathgen.stats.ox.ac.uk/genetics_software/shapeit/shapeit.html), using the 1,000 genomes phase I reference panel (released in June 2014, all samples). QC of imputed probabilities was conducted in QCTOOL 1.4 (http://www.well.ox.ac.uk/~gav/qctool/). Imputed SNPs were excluded if MAF <1%, HWE test *p*-values ≤10^−6^, or an INFO metric <0.8. SNP coordinates are given according to hg19.

### Statistical analyses of genotype data (GWAS)

GWAS for MDD were conducted separately on the two MDD cohorts, recMDD and BoMa. The GWAS for height was carried out using the population-based cohorts of KORA S3 and S4. PLINK was used for these GWAS. Multidimensional scaling (MDS) on the identity-by-state matrix was conducted separately on each GWAS cohort to calculate the ancestry components. For height, the number of consecutive MDS components cumulatively explaining at least 80% of the genetic variance were selected as covariates (*n* = 8) and for MDD, the number of MDS components was selected based on Cattell's scree test (*n* = 3). Sex, age, and MDS components were used as covariates in logistic or linear regression. Data sets were combined using a fixed-effects meta-analysis in METAL (http://csg.sph.umich.edu/abecasis/metal/). The same covariates were retained for analysis with the DeepWAS method. For MS, the published GWAS results were used [14].

### Prediction of regulatory effects using DeepSEA

We employed DeepSEA [8], a deep learning model, to determine the SNPs that potentially play an important role in human traits or diseases by acting through the alteration of regulatory elements. DeepSEA predicts the binding effect of chromatin features to a given sequence of 1,000 bp, which has been shown to significantly improve the model performance compared to smaller window sizes (for details please see [8]). For a specific SNP this is done for both SNP alleles. DeepSEA reaches test set median AUCs of 0.899 for transcription factors, 0.862 for DNase I-hypersensitive sites, and 0.811 for histone marks. All the 919 DeepSEA chromatin features representing regulatory information derived from the profiles of the ENCODE project [9] were considered as FUs (S1 Table). These units covered combinations of 201 different experimental annotations of epigenetically relevant information. This data included 690 TF binding profiles for 160 different TFs, 125 Dnase I hypersensitive site (DHS) profiles, and 104 histone mark profiles across 31 cell lines and 17 treatment conditions. The pre-trained DeepSEA network (v0.94) was downloaded from http://deepsea.princeton.edu/help/ and the predictions and corresponding significance values of the regulatory effects, so-called *e*-values, of the set of all SNPs from three GWAS data sets were generated using an NVIDIA GeForce GTX TITAN X graphics processing unit (GPU) (Maxwell). Significance was assessed by the method proposed by Zhou & Troyanskaya [8], which uses one million random SNPs from the 1,000 genomes project [23] as a background distribution to calculate e-values for each FU, by assessing the proportion of random variants with a bigger effect than that of observed variants. We applied an *e*-value cutoff of 5×10^−5^, to only take the SNPs associated with at least one FU into consideration (e.g., rs1035271 in GM12878:MEF2C). We refer to this set of SNPs as having a predicted regulatory effect.

### DeepWAS

#### Penalized regression models

Compared to the classical GWAS approaches where the trait of interest (*y*) is regressed separately on each SNP *j* (*X*_*j*_), the regularized polygenic regression approaches provide an alternative way to model the joint effect of a set *S*_*k*_ of SNPs (*jϵS*_*k*_). For the present proof of concept study of DeepWAS, we obtained *k = 1…*919 sets *S*_*k*_ of SNPs per functional unit FU from DeepSEA predictions. In order to identify both the most likely mechanism of action for a trait or disease and related regulatory SNPs jointly, we fitted L1-regularized logistic or linear regression (LASSO) with stability selection per FU, *i*.*e*., 919 separate regression models. For simplicity, we further describe only one single FU model of index *k* without subscripting, for example, the estimated coefficients *β*, which are separately estimated per model *k*. Linear models fitted to datasets with a continuous response *y*, like height, are denoted as:

$$y=\underset{j\epsilon S_{k}}{\sum }\left(\beta_{j}^{snp}{\text{X}}_{j}\right)+\beta^{sex}sex+\beta^{age}age+\beta^{cohort}cohort+\overset{n}{\underset{l=1}{\sum }}\left(\beta_{l}^{mds}{\text{mds}}_{l}\right)+\beta_{0}+\epsilon ,$$

where X_*ij*_ represent the SNP matrix of *i* individuals and *j* SNPs and *sex*, *age*, *cohort* and *mds* are vectors with index *i*. The genotypes in the *X* matrix is encoded using the dosage representation of each SNP in an additive model, such that the final encoding of a SNP is *X*_*ij*_ = 2 × *P*(*AA*_*ij*_) + *P*(*Aa*_*ij*_), where *P*(*AA*_*ij*_) and *P*(*Aa*_*ij*_) are probabilities for being homozygous and heterozygous for the minor allele, respectively, for individual *i* and SNP *j*. *X*_*ij*_ is, therefore, a continuous value between 0 and 2. In addition to the SNP predictors, we used sex (*sex*, binary), age (*age*, continuous), a cohort dummy index (*cohort*), and MDS ancestry components (*mds*_*l*_, where *l* represents the MDS ancestry component index, continuous) as covariates. The response vector *y* represents the disease or trait status of interest. In each of the *k* LASSO models, only the SNPs *j* that significantly affect a specific chromatin feature in a specific cell line were included (*jϵS*_*k*_). This is represented in the equation by the summation over the elements of *S*_*k*_, which represents the set of SNPs *j* that has an impact on the FU *S*_*k*_. For logistic regression models, the right-hand side of the linear regression model, without the *ϵ* noise term, models the probability of *y* to be 1 (for binary outcomes), using the binomial distribution function to link it to the binary outcome.

All model parameters (*β*) of the linear regression for a continuous phenotype were optimized with L1 regularization, where the *λ* parameter represents the strength of the regularization. We fitted LASSO models using *glmnet* (https://cran.r-project.org/web/packages/glmnet/index.html) and *stabs* (https://cran.r-project.org/web/packages/stabs/index.html) *R* packages. The stability selection method provides a robust feature selection by taking the uncertainty of feature selection into account, using subsets or bootstrapped subsamples of data sets. The regularization parameter *λ* is determined within the stability selection procedure, based on a provided probability cutoff and per-family error rate (PFER) values [47]. Note that, unlike the family-wise error rate and false discovery rate, which define a probability and an expected proportion, respectively, PFER defines the expected number of false positives and hence can be above 1.0. In total, *n* = 100 subsample replicates were used for each model fit with a subsample size of ⌊*n*/2⌋. The probability cutoff represents how frequently a variable must be selected in LASSO models fitted to these replicates in order to be called a dSNP (an example is shown in S6 Fig). For more details of the relationship between the *λ* parameter, the probability cutoff, and PFER values please see [48].

We decided to use LASSO regression instead of Elastic Net (EN), as the correlation between regulatory SNPs was low, *i*.*e*., of the 36,409 SNPs that were predicted to be regulatory for the imputed, high-quality MS SNP set, 26,155 (72%) were uncorrelated (*r*^*2*^<0.2, assessed via pairwise LD calculations using a window size of 50 SNPs and a shift parameter of 5), with a mean *r*^*2*^ per FU ranging from 0.001 to 0.3. As already reported in [49], LASSO and EN do not differ significantly in their SNP selection for weakly correlated SNPs. Within the MS regulatory SNP set, almost all SNPs identified by LASSO were also found with EN (96–99% overlap, see S5 Fig), which was also the case for the selected FUs. Furthermore, the fine-tuning of the EN meta parameter L2 (alpha) is more difficult and time-consuming, increasing the danger of overfitting (for more details please see [49]).

#### DeepWAS application

DeepWAS was conducted on the KKNMS dataset for MS, on the MDDC dataset for MDD, and on the KORA dataset for height, see Table 1. Sex, age, cohort membership, and selected MDS ancestry components were used as covariates in DeepWAS. “*stabsel*” function from the *stabs* R package was used to identify significant trait associations (dSNPs). “cutoff”, “PFER” and “fitfun” parameters of “*stabsel”* function, which denote the selection probability cutoff, per-family error rate and LASSO fitting method were set to 0.7, 1.0 and “glmnet.lasso”, respectively. We identified independent SNPs that are dSNPs and independent from each other by pruning SNPs using the PLINK—indep-pairwise command (settings: window size 50 kb, step size 5, *r*^*2*^ > 0.5).

### DeepWAS vs. GWAS

KKNMS genotype data was randomly split into a training (80%, *n* = 12,227 individuals) and a test set (20%, *n* = 3,056 individuals). The AUC values were evaluated based on the test set and measured by using the *“prediction”* and *“performance”*functions of the *ROCR* R package. For DeepWAS, we fitted a LASSO model for each FU (n = 444 FUs) based on the training set including all covariates. We tuned λ by a ten-fold cross-validation (CV) method using the “*cv*.*glmnet*” function from the *glmnet* R package. The “lambda.min” parameter, which denotes the value of λ that gives minimum mean cross-validated error, was extracted and used for prediction. For GWAS, we fitted a logistic regression model including all covariates mentioned above using the *“glm”* function for each genome-wide significant KKNMS GWAS hit (n = 606 SNPs).

### Functional annotation of dSNPs

#### Annotation of dSNPs with ENCODE tissue categories

ENCODE cell type information was downloaded from https://genome.ucsc.edu/encode/cellTypes.html and tissue categories were extracted from the column “tissue”.

#### Roadmap cis-regulatory elements (ChromHMM)

*Cis*-regulatory elements identified by the Roadmap Epigenomics Project [10] were downloaded as segmentation files of core 15-state ChromHMM model for 111 epigenomes from the Roadmap epigenomics web portal (http://egg2.wustl.edu/roadmap/data/byFileType/chromhmmSegmentations/ChmmModels/coreMarks/jointModel/final/all.mnemonics.bedFiles.tgz) in BED format. ChromHMM used five core marks (H3K4me3, H3K4me1, H3K36me3, H3K27me3, H3K9me3) from each of the 111 reference epigenomes and learned a set of 15 chromatin state definitions per genomic segment. We overlapped the DeepWAS SNPs with chromatin states based on the exact genomic position. All roadmap epigenomes were grouped into broader tissue groups, which were used for mapping between DeepWAS and ChromHMM results.

#### Genomic region annotation

DeepWAS SNPs were overlapped with genomic annotation from UCSC for the hg19 genome build using *TxDb*.*Hsapiens*.*UCSC*.*hg19*.*knownGene* (https://bioconductor.org/packages/release/data/annotation/html/TxDb.Hsapiens.UCSC.hg19.knownGene.html) and *ChIPseeker* (https://www.bioconductor.org/packages/release/bioc/html/ChIPseeker.html) Bioconductor *R* packages.

Tissue enrichment of dSNPs with SNPsea and FANTOM5 CAGE data. Tissue enrichment was performed with the command line interface of *SNPsea* version 1.0.3 (https://github.com/slowkow/snpsea/). All data and annotation files were the default ones provided by *SNPsea* (tissue-specific gene expression from ~400 cell types), namely the *FANTOM2014*.*gct*.*gz* gene expression file was used with default *NCBIgenes2013*.*bed*.*gz*, *TGP2011*.*bed*.*gz*, *Lango2010*.*txt*.*gz* values for “gene-intervals”, “snp-intervals”, and “null-snps” options, respectively.

#### Pathogenic annotation

Pathogenic annotation was performed using the CADD webserver.

### DNA methylation data

For a subset of the MDDC cohort (n = 166 recMDD cases), genomic DNA was extracted from whole blood using the Gentra Puregene Blood Kit (QIAGEN). DNA quality and quantity of both was assessed with the NanoDrop 2000 Spectrophotometer (Thermo Scientific) and Quant-iT Picogreen (Invitrogen). Genomic DNA was bisulfite converted using the Zymo EZ-96 DNA Methylation Kit (Zymo Research) and DNA methylation levels were assessed for >480,000 CpG sites using the Illumina HumanMethylation450K BeadChips. Hybridization and processing were performed according to the manufacturer’s instructions. QC of methylation data, including intensity readouts, filtering (detection *p*-value >0.01 in at least 75% of the samples), cellular composition estimated using *FlowSorted*.*Blood*.*450k* data and “estimateCellCounts” function, as well as beta calculation (“getBeta” function) were done using the *minfi* Bioconductor *R* package (https://bioconductor.org/packages/release/bioc/html/minfi.html). CpG sites on sex chromosomes, CpG site probes found to have SNPs at the CpG site itself or in the single-base extension site with a MAF ≥1% in the 1,000 genomes project EUR population and non-specific binding CpG site probes according to [50] were removed. We performed a re-alignment of the CpG site probe sequences using *Bismark* (https://www.bioinformatics.babraham.ac.uk/projects/bismark/). This yielded 425,883 CpG sites for further analysis. The data were then normalized using functional normalization (“preprocessFunnorm” function in *minfi*). Technical batch effects were identified by inspecting the association of the first principal components of the methylation levels with plate and plate position. The data were then adjusted using “*ComBat”* function of the Bioconductor *R* package *sva* (https://bioconductor.org/packages/release/bioc/html/sva.html). CpG coordinates are given according to hg19.

DNA methylation data was also available for a subset of the KORA study (*n* = 1,802 F4 individuals). Here, DNA methylation was measured with the Illumina HumanMethylation450K BeadChips. Sample preparation and measurement have been described previously [51]. Intensity values were extracted from the *idat* files using *minfi*, with subsequent background correction performed with *lumi* Bioconductor *R* package (https://bioconductor.org/packages/release/bioc/html/lumi.html). CpG site probes with a detection *p*-value >0.01 or summarized by less than 3 functional beads were set to missing. A sample-wise call rate of 80% was applied, and color bias adjustment using smooth quantile normalization was performed. Finally, beta mixture quantile normalization was performed on the probes (“BMIQ” function in Bioconductor *R* package *watermelon* [https://bioconductor.org/packages/release/bioc/html/wateRmelon.html]) to correct for the Inf I/Inf II distribution shift.

### Gene expression data

Gene expression analysis of a subset of the KKNMS cohort (*n* = 319 DE1 MS cases) was performed using lllumina HT-12 v4 Expression BeadChips, as described in [14]. For a subset of the KORA cohort (*n* = 1,002 F4 individuals) gene expression profiling was performed using the Illumina HT-12 v3 Expression BeadChips and described previously in [52].

### Statistical analysis of gene expression and methylation data

For the MDDC cohort, linear regression models were fit for each CpG site to test the relationship between the whole blood DNA methylation (beta values) and proximal SNP genotype (in dosage format) within 1Mb up- or downstream of the SNP using the *R* package *MatrixEQTL* (https://cran.r-project.org/web/packages/MatrixEQTL/index.html), in order to detect *cis*-meQTLs. Sex, blood cell counts, and MDS ancestry components to correct for possible admixture effects were included as covariates. Significance after multiple testing was adjusted using a false discovery rate (FDR) of 5%.

For the whole blood *cis*-meQTL analysis in the KORA cohort we used the OmicAbel software (https://github.com/GenABEL-Project/OmicABEL) with age, sex, and blood cell counts as covariates. A total of 1,731 individuals had valid methylation and genetic data available. Significance was defined using Bonferroni correction at a *p*-value of 1×10^−14^. To examine the relationship between proximal genetic variation and gene expression, *i*.*e*. *cis*-eQTLs, in KORA (*n* = 711 individuals with valid genetic and expression data), we first derived residuals for gene transcript expression using linear regression of log_2_-transformed gene transcript levels against sex, age, the RNA integrity number, RNA amplification plate, and sample storage time. Expression residuals were then used as outcome variables in a linear regression model with SNP dosage as the independent variable. Data analysis was performed using *MatrixEQTL* and significant *cis*-eQTLs were filtered at an FDR of 5%.

For the whole blood *cis*-eQTL analysis in the KKNMS cohort, we used *MatrixEQTL* with sex, age, blood cell counts estimated using *R* package *CellCODE* (http://www.pitt.edu/~mchikina/CellCODE/), and MDS ancestry components as covariates. Significance after multiple testing was adjusted using an FDR of 5%.

Using published QTL data from BIOS whole peripheral blood DNA of 3,841 and mRNA of 2,116 healthy samples [28] (eQTL, meQTL, expression Quantitative Trait Methylation, *i*.*e*. eQTM), downloaded from http://genenetwork.nl/biosqtlbrowser, CMC eQTLs [29] downloaded from https://www.synapse.org/#!Synapse:syn4622659, and GTEx eQTLs [30] downloaded from https://gtexportal.org/home/datasets, we were able to intersect our dSNPs and their LD proxies (*r*^*2*^≥0.5) with transcriptionally relevant data. The eQTLs obtained from GTEx were filtered on gene *p*<0.05 and eQTLs obtained from BIOS and CMC were filtered on FDR <0.05. We used permutation tests to determine if dSNPs or their LD proxies were enriched in BIOS QTLs. For each phenotype, we compared the overlap of dSNPs and BIOS QTL SNPs to the average overlap from 1,000 equally-sized sets of regulatory SNPs and BIOS QTL SNPs.

### QTL network

For the visualization of dSNP-QTL interactions, we set up a *Neo4j* v3.4.0 instance (https://neo4j.com). All DeepWAS results were inserted into the database. The graph structure consisted of genes, transcription factors, CpGs, SNPs, cell lines, and tissues that were connected to each other through dummy nodes representing each dSNP. Dummy nodes are especially important in cases where a dSNP is predicted to be active in more than one FU, e.g., TF1:CL1 and TF2:CL2. In this case, connecting these four elements directly to the dSNP leads to ambiguity about the FU, which can be misinterpreted as TF1:CL2 or TF2:CL1, since the information of the FU would be lost. Using dummy nodes avoids this confusion by providing a SNP-FU link.

## Supporting information

S1 Fig Heatmaps of selected chromatin features vs. cell line for MDD and height. The heatmaps show the number of selected chromatin features vs. cell line for (**A**) MDD and **(B**) height dSNPs. Chromatin features are limited to be at least present in two distinct cell lines.
(PDF)

S2 Fig MDD DeepWAS results involving the TF MEF2C and its functional annotation. **(A**) Graph-based QTL Network visualization of the DeepWAS results involving the TF MEF2C, which itself is one of the top associated PGC GWAS loci. Two dSNPs are jointly associated with MDD and belong the same FU: MEF2C:GM12878. Edges represent the association relation of dSNPs, chromatin features with or without treatment, cell lines, top-level tissue group, CpGs, and genes. Edges of the FU MEF2C:GM12878 are colored in red. Circular shades mark the corresponding genes or CpGs with are plotted in B and C. (**B**) Box plot of GTEx frontal cortex eQTL data showing relationship between *SPIDR* gene expression and dSNP rs7839671. (**C**) Boxplot of recMDD meQTL data illustrating relationship between cg01650371 methylation and rs10099827 genotype in recMDD samples. Variant rs10099827 is a proxy of dSNP rs7839671 (*r*^**2**^ = 0.8). DSNP rs7839671 exhibits a meQTL effect on the same CpG and was excluded from the original meQTL. analysis, as it is 571 kb away from the CpG site (meQTL distance cutoff≤250 kb).
(PDF)

S3 Fig MS GWAS and DeepWAS results for rs2273030 and its functional annotation. **(A**) Locus-specific Manhattan plot of the dSNP rs2273030 that is a sub-threshold GWAS SNP for MS. The plot is produced using LocusZoom (https://github.com/statgen/locuszoom). with EUR samples of the 1,000 genomes November 2014 reference panel on the hg19 build. Dots represent GWAS *p*-values and the color of dots indicates LD with the lead variant, grey dots have LD *r*^*2*^ missing. (**B**) MS-specific three-way QTL interaction network generated by using a graph database and highlighting only the dSNPs with eQTL and meQTL effects that also harbor an eQTM. Edges represent the association relation of dSNPs, chromatin features with or without treatment, cell lines, top-level tissue group, CpGs, and genes.
(PDF)

S4 Fig Three-way QTL interaction network. (**A**) Height-specific three-way QTL interaction network highlighting dSNP rs7146599 on chromosome 14 as one of the moderators of height in eleven cell lines. It affects a cascade of chromatin features (n = 8) and shows meQTL and eQTL effects that, at the same time, harbor an eQTM. The CpG site and the dSNP thus affect the transcriptional level of the same genes. In addition, the network includes rs2871960 on chromosome 3, linked to the *ZBTB38* locus and correlated with multiple CpG sites. Edges represent the association relation of dSNPs, chromatin features with or without treatment, cell lines, top-level tissue group, CpGs, and genes. (**B**) MDD-specific three-way QTL interaction network generated by using a graph database and highlighting only the dSNPs with eQTL and meQTL effects that also harbor an eQTM. It shows that the MDD-specific dSNPs rs163105 on chromosome 5 changes the expression of *SKIV2L2* (also known as *MTR4*). Edges represent the association relation of dSNPs, chromatin features with or without treatment, cell lines, top-level tissue group, CpGs, and genes.
(PDF)

S5 Fig Comparison of LASSO vs. Elastic Net. The LASSO picked out the smallest number of SNPs (n = 164 SNPs in 708 FU). The EN with penalty weight α = 0.1 (EN01) selected 169 SNPs in 710 FU, EN with α = 0.5 (EN05) selected 174 SNPs in 709 FU and EN with α = 0.75 (EN075) selected 169 SNPs in 706 FU. The single LASSO regression (SLR) (all regulatory MS SNPs without grouping to FUs) identified 19 SNPs showing a significant association with MS, these SNPs were also part of the sets of selected SNPs by either LASSO or EN.
(PDF)

S6 Fig LASSO stability selection results for FU MEF2C-GM12878. The y-axis indicates number of boosting iterations, the x-axis indicates the stability selection probability, and the horizontal line correspond the 0.7 probability threshold.
(PDF)

S1 Table List of all functional units. (XLSX)

S2 Table DeepWAS results for MS and functional annotation. (XLSX)

S3 Table DeepWAS results for MDD and functional annotation. (XLSX)

S4 Table DeepWAS results for height and functional annotation. (XLSX)
