# Supplementary figures and images for: DeepWAS: Multivariate genotype-phenotype associations by directly integrating regulatory information using deep learning
Source: PLoS Comput Biol. 2020 Feb 3;16(2):e1007616. doi: 10.1371/journal.pcbi.1007616 (PMC7043350; doi:10.1371/journal.pcbi.1007616)

A

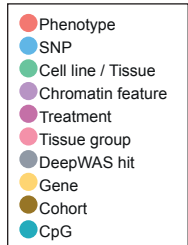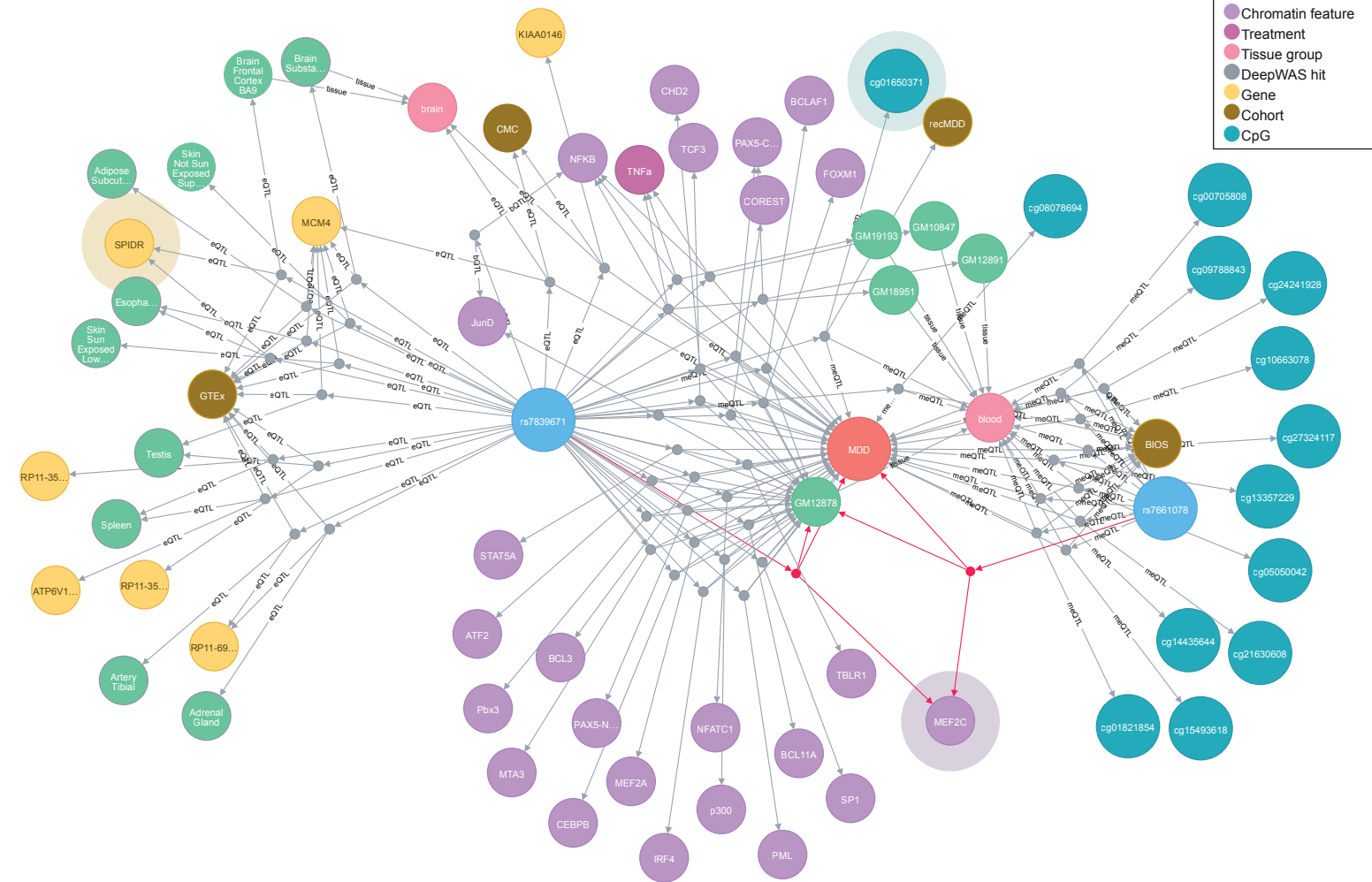

B

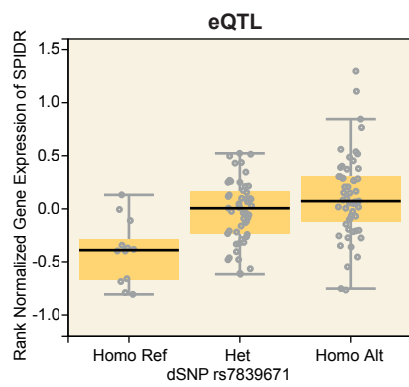

C

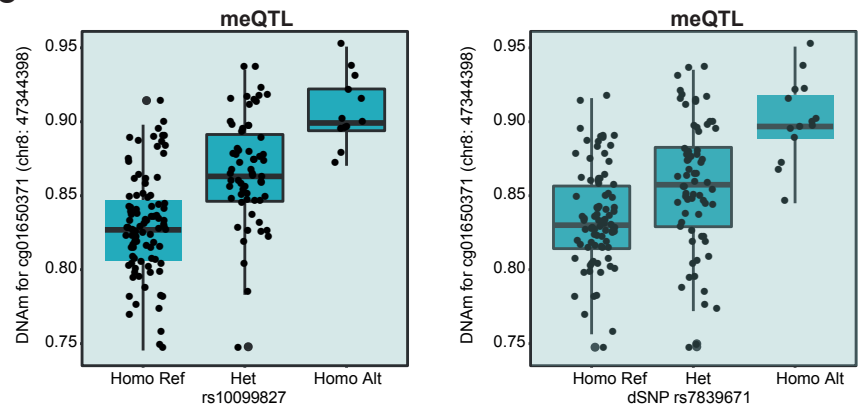

Supplement: S2 Fig — (A) Graph-based QTL Network visualization of the DeepWAS results involving the TF MEF2C, which itself is one of the top associated PGC GWAS loci. Two dSNPs are jointly associated with MDD and belong the same FU: MEF2C:GM12878. Edges represent the association relation of dSNPs, chromatin features with or without treatment, cell lines, top-level tissue group, CpGs, and genes. Edges of the FU MEF2C:GM12878 are colored in red. Circular shades mark the corresponding genes or CpGs with are plotted in B and C. (B) Box plot of GTEx frontal cortex eQTL data showing relationship between SPIDR gene expression and dSNP rs7839671. (C) Boxplot of recMDD meQTL data illustrating relationship between cg01650371 methylation and rs10099827 genotype in recMDD samples. Variant rs10099827 is a proxy of dSNP rs7839671 (r2 = 0.8). DSNP rs7839671 exhibits a meQTL effect on the same CpG and was excluded from the original meQTL. analysis, as it is 571 kb away from the CpG site (meQTL distance cutoff≤250 kb). (PDF) [file pcbi.1007616.s002.pdf]

**B**

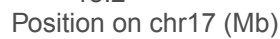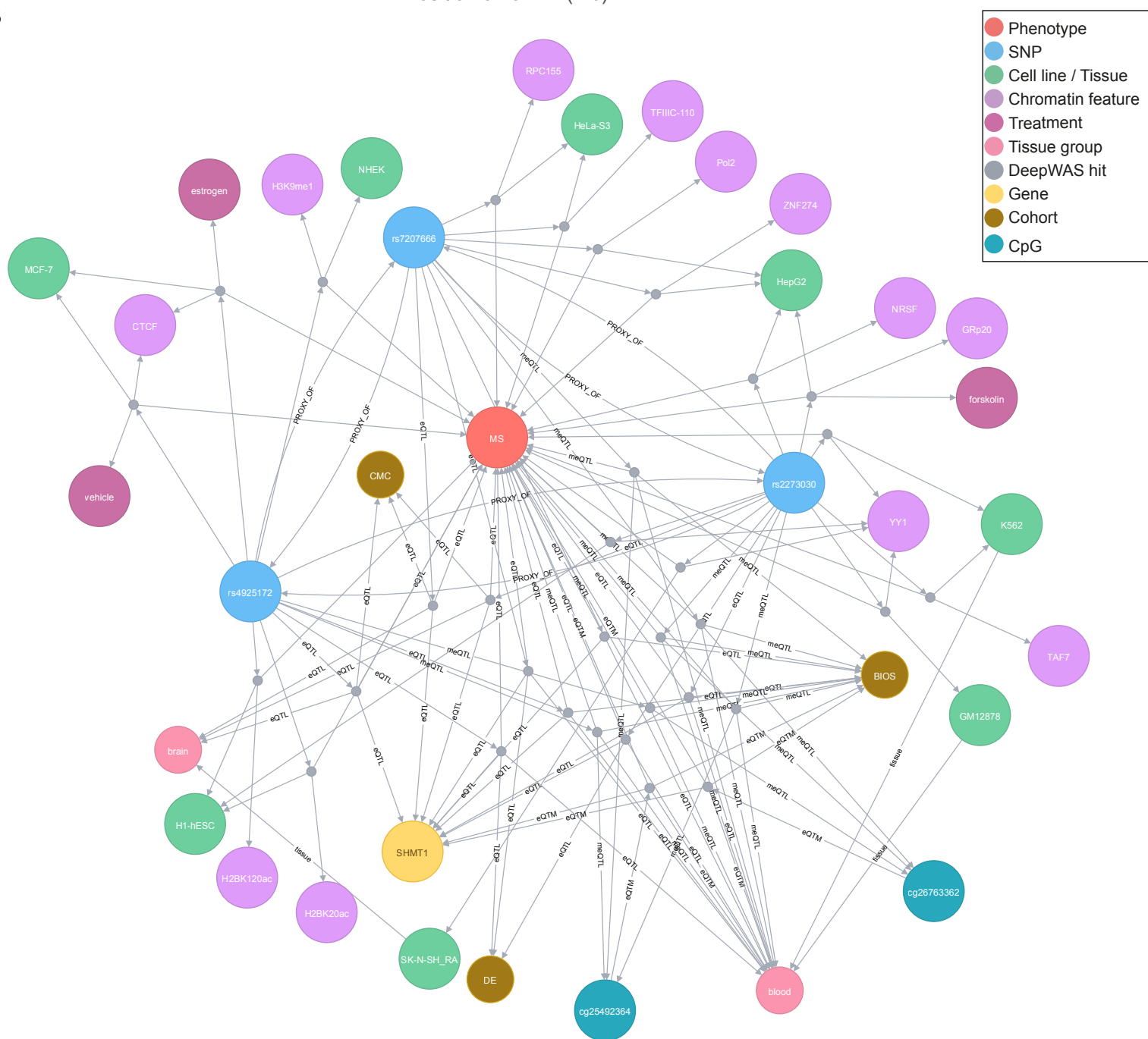

Supplement: S3 Fig — (A) Locus-specific Manhattan plot of the dSNP rs2273030 that is a sub-threshold GWAS SNP for MS. The plot is produced using LocusZoom (https://github.com/statgen/locuszoom). with EUR samples of the 1,000 genomes November 2014 reference panel on the hg19 build. Dots represent GWAS p-values and the color of dots indicates LD with the lead variant, grey dots have LD r2 missing. (B) MS-specific three-way QTL interaction network generated by using a graph database and highlighting only the dSNPs with eQTL and meQTL effects that also harbor an eQTM. Edges represent the association relation of dSNPs, chromatin features with or without treatment, cell lines, top-level tissue group, CpGs, and genes. (PDF) [file pcbi.1007616.s003.pdf]

**A**

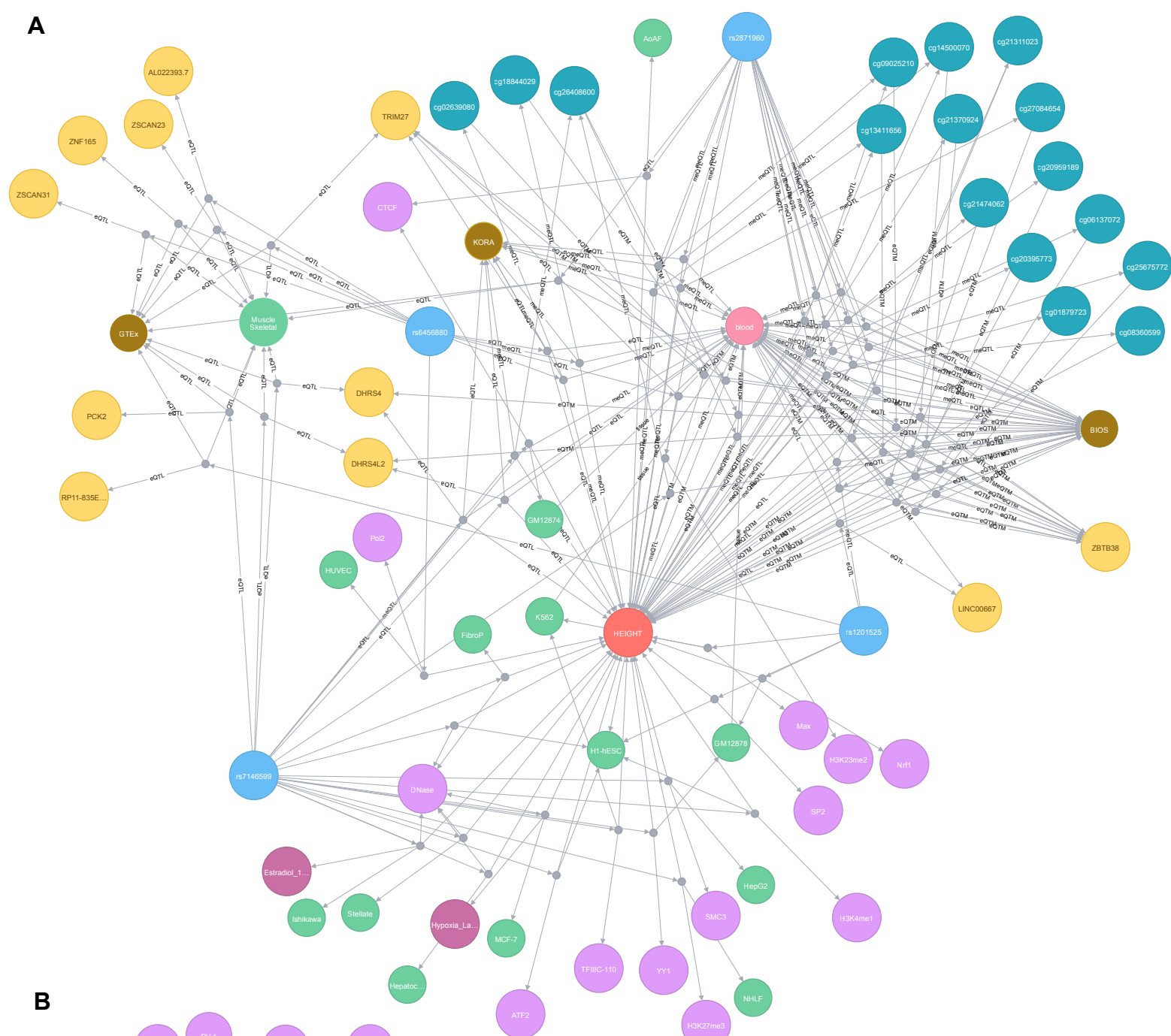

# B

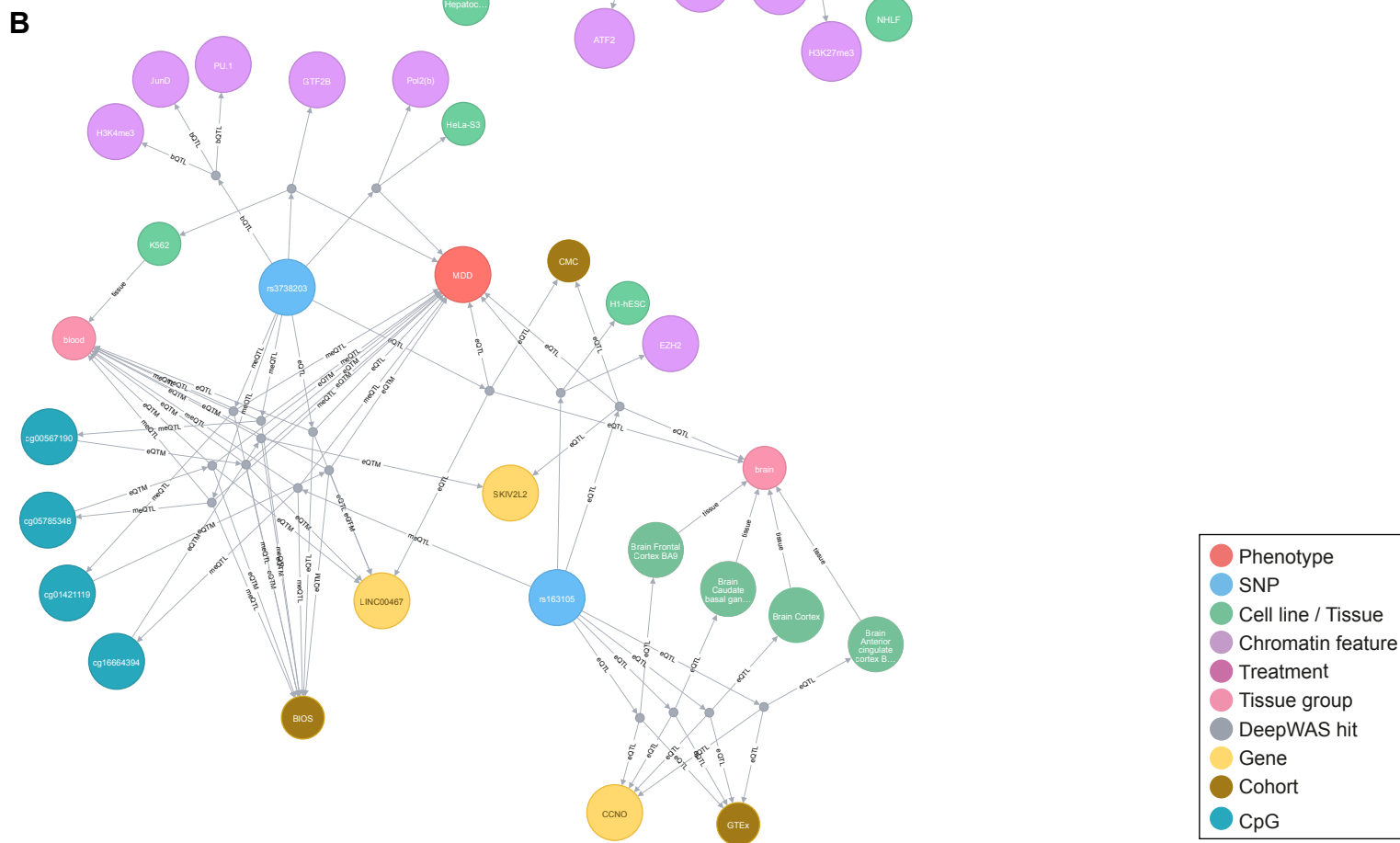

Supplement: S4 Fig — (A) Height-specific three-way QTL interaction network highlighting dSNP rs7146599 on chromosome 14 as one of the moderators of height in eleven cell lines. It affects a cascade of chromatin features (n = 8) and shows meQTL and eQTL effects that, at the same time, harbor an eQTM. The CpG site and the dSNP thus affect the transcriptional level of the same genes. In addition, the network includes rs2871960 on chromosome 3, linked to the ZBTB38 locus and correlated with multiple CpG sites. Edges represent the association relation of dSNPs, chromatin features with or without treatment, cell lines, top-level tissue group, CpGs, and genes. (B) MDD-specific three-way QTL interaction network generated by using a graph database and highlighting only the dSNPs with eQTL and meQTL effects that also harbor an eQTM. It shows that the MDD-specific dSNPs rs163105 on chromosome 5 changes the expression of SKIV2L2 (also known as MTR4). Edges represent the association relation of dSNPs, chromatin features with or without treatment, cell lines, top-level tissue group, CpGs, and genes. (PDF) [file pcbi.1007616.s004.pdf]

**A**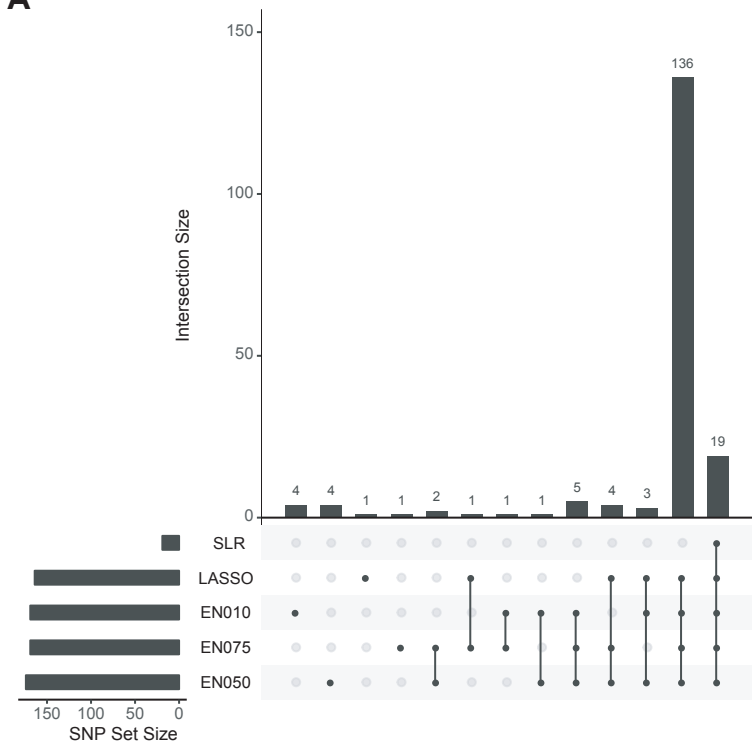**B**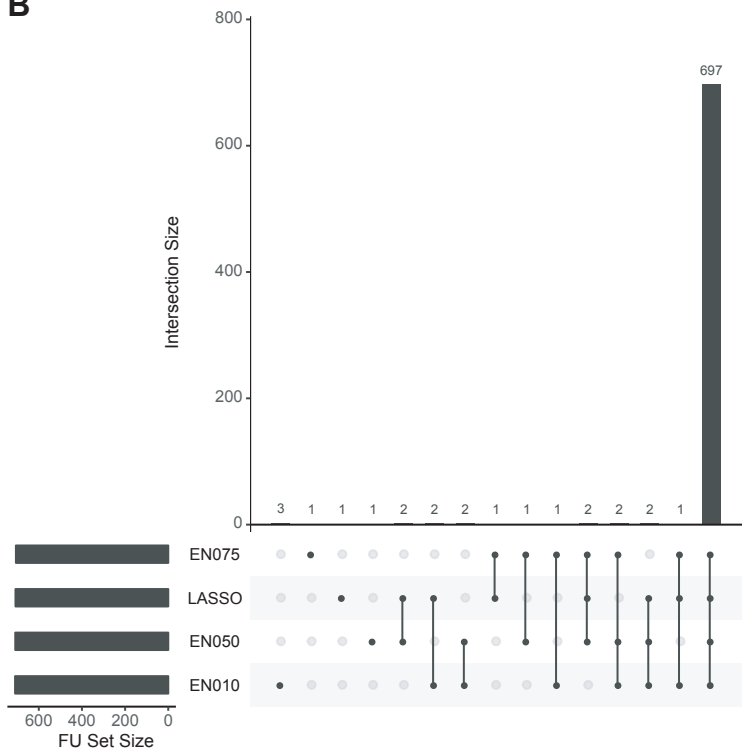

Supplement: S5 Fig — The LASSO picked out the smallest number of SNPs (n = 164 SNPs in 708 FU). The EN with penalty weight α = 0.1 (EN01) selected 169 SNPs in 710 FU, EN with α = 0.5 (EN05) selected 174 SNPs in 709 FU and EN with α = 0.75 (EN075) selected 169 SNPs in 706 FU. The single LASSO regression (SLR) (all regulatory MS SNPs without grouping to FUs) identified 19 SNPs showing a significant association with MS, these SNPs were also part of the sets of selected SNPs by either LASSO or EN. (PDF) [file pcbi.1007616.s005.pdf]

FU: MEF2C-GM12878

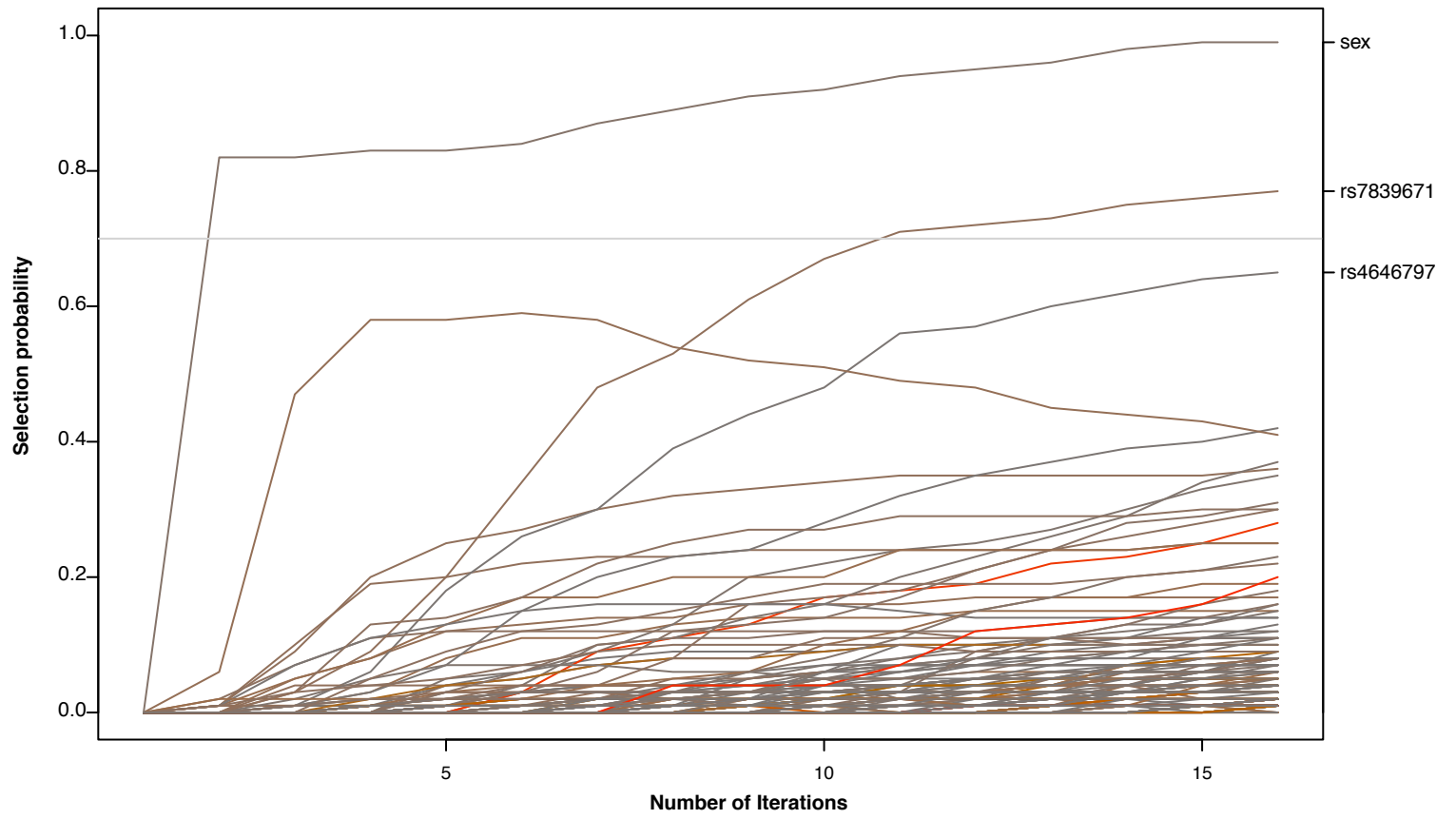

Supplement: S6 Fig — The y-axis indicates number of boosting iterations, the x-axis indicates the stability selection probability, and the horizontal line correspond the 0.7 probability threshold. (PDF) [file pcbi.1007616.s006.pdf]
