# Supplementary material for: DeepWAS: Multivariate genotype-phenotype associations by directly integrating regulatory information using deep learning
Source: PLoS Comput Biol. 2020 Feb 3;16(2):e1007616. doi: 10.1371/journal.pcbi.1007616 (PMC7043350; doi:10.1371/journal.pcbi.1007616)
Supplement: S1 Fig — The heatmaps show the number of selected chromatin features vs. cell line for (A) MDD and (B) height dSNPs. Chromatin features are limited to be at least present in two distinct cell lines. (PDF) [file pcbi.1007616.s001.pdf]

A

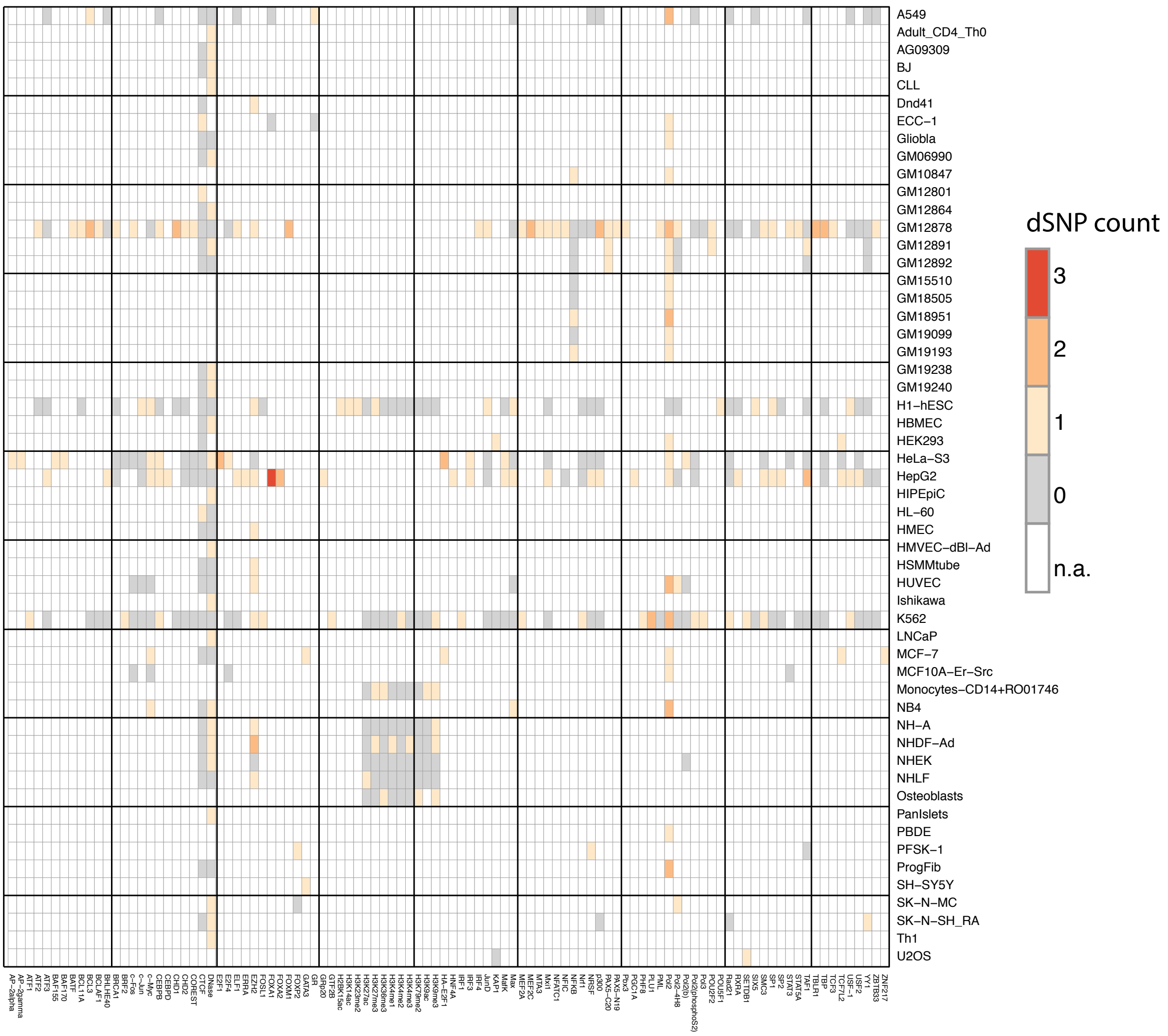
A549
Adult_CD4_Th0
AG09309
BJ
CLL
Dnd41
ECC-1
Gliobla
GM06990
GM10847
GM12801
GM12864
GM12878
GM12891
GM12892
GM15510
GM18505
GM18951
GM19099
GM19193
GM19238
GM19240
H1-hESC
HBMEC
HEK293
HeLa-S3
HepG2
HIPEpiC
HL-60
HMEC
HMVEC-dBl-Ad
HSMMtube
HUVEC
Ishikawa
K562
LNCaP
MCF-7
MCF10A-Er-Src
Monocytes-CD14+RO01746
NB4
NH-A
NHDF-Ad
NHEK
NHLF
Osteoblasts
PanIslets
PBDE
PFSK-1
ProgFib
SH-SY5Y
SK-N-MC
SK-N-SH_RA
Th1
U2OS
dSNP count
3
2
1
0
n.a.

B

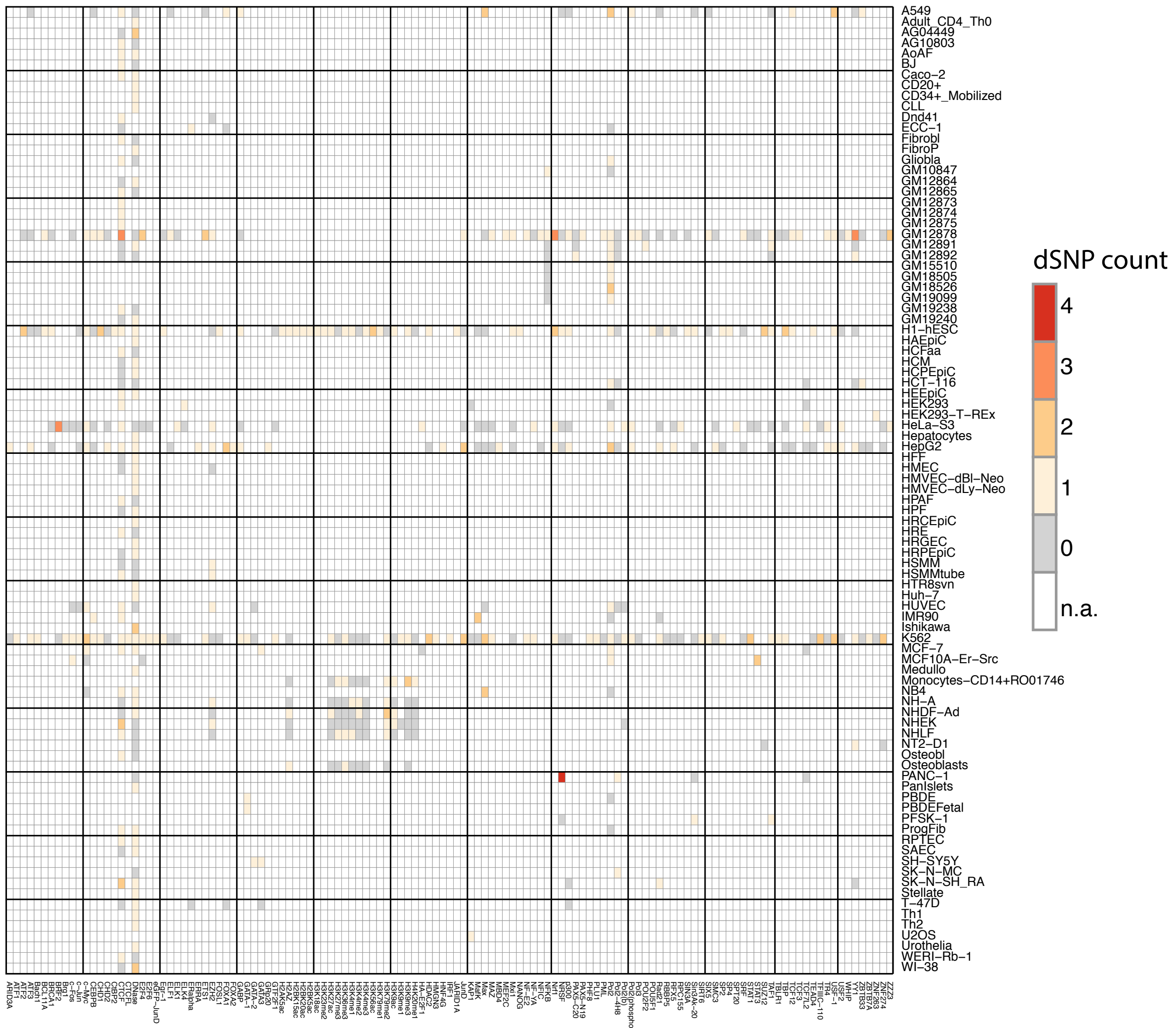
A549
Adult_CD4_Th0
AG04449
AG10803
AoAF
BJ
Caco-2
CD20+
CD34+_Mobilized
CLL
Dnd41
ECC-1
Fibrobl
FibroP
Gliobla
GM10847
GM12864
GM12865
GM12873
GM12874
GM12875
GM12878
GM12891
GM12892
GM15510
GM18505
GM18526
GM19099
GM19238
GM19240
H1-hESC
HAEpiC
HCFaa
HCM
HCPEpiC
HCT-116
HEEpiC
HEK293
HEK293-T-REx
HeLa-S3
Hepatocytes
HepG2
HFF
HMEC
HMVEC-dBl-Neo
HMVEC-dLy-Neo
HPAF
HPF
HRCEpiC
HRE
HRGEC
HRPEpiC
HSMM
HSMMtube
HTR8svn
Huh-7
HUVEC
IMR90
Ishikawa
K562
MCF-7
MCF10A-Er-Src
Medullo
Monocytes-CD14+RO01746
NB4
NH-A
NHDF-Ad
NHEK
NHLF
NT2-D1
Osteobl
Osteoblasts
PANC-1
PanIslets
PBDE
PBDEFetal
PFSK-1
ProgFib
RPTEC
SAEC
SH-SY5Y
SK-N-MC
SK-N-SH_RA
Stellate
T-47D
Th1
Th2
U2OS
Urothelia
WERI-Rb-1
WI-38
dSNP count
4
3
2
1
0
n.a.
